# Clifti-GPT: privacy-preserving federated fine-tuning and transferable inference of foundation models on clinical single-cell data

**DOI:** 10.1186/s13040-026-00582-w

**Published:** 2026-08-05

**Authors:** Mohammad Bakhtiari, Maria Louise Elkjaer, Ali Oğuz Can, Fabian Theis, Mhaned Oubounyt, Jan Baumbach

**Affiliations:** 1 Institute for Computational Systems Biomedicine, University of Hamburg, Hamburg, Germany; 2 Institute for Inflammation Research, Center for Rheumatology and Spine Diseases, Copenhagen University Hospital, Rigshospitalet, Copenhagen, Denmark; 3 Institute of Computational Biology, Helmholtz Center, Munich, Germany; 4 TUM School of Life Sciences, Technical University of Munich, Munich, Germany; 5 TUM, School of Computation, Information and Technology, Technical University of Munich, Munich, Germany; 6 Department of Mathematics and Computer Science, University of Southern Denmark, Odense, Denmark

**Keywords:** Federated learning, Privacy-preserving machine learning, Single-cell RNA sequencing, Foundation models, Secure multi-party computation

## Abstract

Foundation models have demonstrated immense value for scRNA-seq analysis, but their fine-tuning or inference on heterogeneous, privacy-sensitive clinical cohorts is governed by strict data protection policies, which often prohibit centralization. We introduce Clifti-GPT, a privacy-preserving federated framework based on secure multi-party computation (SMPC) that enables collaborative model training and transferable inference, where zero-shot predictions are performed across decentralized clinical repositories by securely aggregating local statistics rather than transferring data embeddings, without sharing patient data, clinical-level statistics, or models. Built upon the scGPT foundation model, Clifti-GPT achieves performance within 4% of centralized scGPT baselines in accuracy, precision, recall, and macro-F1 for cell type classification and reference mapping across six datasets. Furthermore, it demonstrates rapid convergence in terms of communication rounds, reaching 99% of centralized performance on cell type classification in at most two federated rounds on two evaluated datasets, and scales robustly to 30 clients with less than 2% accuracy loss on a large-scale federated cell type classification setting. Our analysis shows that batch effects impact both Clifti-GPT and centralized baseline, while correction leads to similar results across evaluation metrics in heterogeneous settings for both models. Together, these results indicate that Clifti-GPT enables effective fine-tuning and application of single-cell foundation models across distributed clinical datasets in a manner that is GDPR-compatible by design and addresses real-world privacy and institutional data-governance requirements.

**Supplementary Information:**

The online version contains supplementary material available at 10.1186/s13040-026-00582-w.

## Introduction

Single-cell RNA sequencing (scRNA-seq) has significantly advanced our understanding of cellular heterogeneity, cells lineage tracking, and disease mechanisms, enabling comprehensive data atlases like the Human Cell Atlas [1]. The rapid expansion of scRNA-seq data necessitates advanced methods for managing and analyzing it to gain meaningful biological insights effectively. Foundation models, such as those built on the self-attention transformer architectures, offer a promising solution. These models, pretrained on large-scale and diverse datasets, have demonstrated strong performance across multiple domains, including biological applications such as Enformer [2], which predicts gene expression directly from DNA sequence by modeling long-range regulatory interactions. By leveraging generative pretraining, foundation models have been shown to adapt effectively to a wide range of downstream tasks, often outperforming task-specific models, making them ideal for the complex and expansive datasets in single-cell research. This approach has the potential to unify and enhance current fragmented machine-learning methods in scRNA-seq (cell typing, trajectory analysis, cell-cell communications, perturbation effects) within one framework, enabling more comprehensive and scalable analyses.

The progression of foundation models for scRNA-seq has rapidly advanced, opening new avenues for leveraging large-scale data in biological research. Early models like scBERT [3] and Geneformer [4] introduced the concept of pretraining on vast single-cell datasets, enabling the transfer of learned representations to specific downstream tasks such as cell type annotation and gene function prediction. Building on these foundations, the emergence of models like scGPT [5], UCE [6], and scFoundation [7] has further solidified this paradigm. scGPT, as a foundation model specifically designed for scRNA-seq data, leverages self-attention transformer architectures and has demonstrated strong performance across a wide range of single-cell applications. scGPT is pre-trained on the extensive CELLxGENE scRNA-seq datasets (https://cellxgene.cziscience.com/), which encompasses a wide range of cell types, tissues, species, and conditions, providing a comprehensive resource for learning intricate patterns across different biological contexts. This large-scale pre-training enables scGPT to excel in tasks such as multi-omics data integration, cell type annotation, and gene function prediction. Similarly, scFoundation offers a scalable framework for querying and analyzing diverse cell atlases, while models like SCimilarity [8] exemplify the utility of foundation models in identifying cellular commonalities across various conditions, such as cancer or immune disorders. Additionally, nicheFormer [9] introduces a hybrid transformer-graph architecture tailored for sparse, high-dimensional single-cell data, enhancing representation learning in both dissociated and spatial contexts. Complementing this, scGPT-Spatial [10] integrates spatial transcriptomics modalities by combining positional encoding with gene expression embeddings. Importantly, it retains full compatibility with dissociated scRNA-seq data, thereby unifying spatial and non-spatial analyses within a single foundation framework. Collectively, these models reflect a broader shift toward foundation-model–based approaches in scRNA-seq analysis, enabling more unified and scalable workflows that extend beyond traditional task-specific methods. In parallel, models like scJoint [11] and scTab [12] have significantly influenced the field by integrating multimodal data and scaling cell type classification across tissues, paving the way for more robust, scalable foundation models in the single-cell field.

As scRNA-seq enters the age of large-scale foundation models, two privacy and governance challenges demand attention. First, raw or minimally processed single-cell count matrices should not be assumed to be anonymous. Although they do not usually contain explicit identifiers, they can encode high-dimensional molecular signals reflecting donor-specific biology, including genotype–phenotype correlations, eQTL-driven transcriptional states, disease-associated expression patterns, rare cellular populations, treatment-related signatures, and tissue- or cohort-specific molecular profiles. Genetic privacy studies show that identities and sensitive traits can be inferred by linking molecular signals with quasi-identifiers, metadata, or external genomic resources, and recent work further demonstrates that private information can leak from single-cell count matrices [13–16]. When combined with auxiliary genomic, clinical, cohort-level, ancestry, or family-related data, these molecular signals may support donor linkage, disease-status inference, or inference of related sensitive attributes [17]. We therefore treat scRNA-seq matrices as potentially identifiable biomedical data rather than as fully anonymized measurements raising regulatory relevance under GDPR Article 4(1) [18]. Sample annotations can pose an additional and often more immediate identification risk. In clinical single-cell studies, metadata may include donor or sample identifiers, disease status, tissue or anatomical sampling site, treatment condition, cohort or batch membership, institution, and cell-type labels. Some of these variables have small domains or rare categories–such as rare diagnoses, uncommon tissue sources, unusual treatment arms, or site-specific cohorts–and can directly narrow the set of possible donors. Metadata and expression matrices should therefore be viewed as complementary privacy risks in single-cell transcriptomics: metadata can expose sensitive clinical context directly, whereas molecular profiles can become more identifying when linked to sample annotations.

Furthermore, institutional data-governance frameworks impose stringent policies on who may access, analyze, or publish both raw data and derived discoveries, such as novel cell types or mutations, driven by intellectual-property, publication priority, and regulatory compliance considerations [19]. Conventional data sharing or centralized model training conflicts with these governance mandates, risking both patient privacy and institutional liability. Together, these vulnerabilities highlight the need for privacy-preserving and governance-aware analytical workflows. By keeping raw and minimally processed counts on-premises and exchanging only encrypted or aggregated model updates and statistics, such workflows aim to reduce re-identification risk and better align with institutional data-governance requirements without necessarily sacrificing analytical breadth or model performance.

Federated Learning (FL) [20] offers a data-local paradigm by enabling decentralized model training without sharing raw data, while also addressing heterogeneity [21–23] and data-imbalance [24] challenges. FL aligns with data-protection and regulatory requirements by design [19, 25, 26], and its legal and governance implications have been extensively documented in prior works [27]. Moreover, the development of FL platforms like FeatureCloud [28] has facilitated deployment of real-world applications in biomedicine. Despite these advances in federated infrastructure, deep-learning models trained on scRNA-seq remain vulnerable to “interrogation” attacks [13]: Model-inversion can reconstruct individual gene-expression profiles from outputs [29], and gradient-leakage [30] in federated settings can recover sensitive count data from shared weight updates. While standard federated learning preserves data locality, it does not by itself prevent leakage from intermediate updates, embeddings, or model outputs[20, 29, 30]. Privacy-enhancing technologies (PETs) address related but distinct privacy goals and trust assumptions [25, 31]. For instance, de-identification alone may be insufficient for health and molecular data that remain linkable to external information sources [13–17]. Differential privacy (DP) can mitigate certain output-leakage risks but may introduce privacy–utility trade-offs, whereas homomorphic encryption (HE) and trusted execution environments (TEEs) provide alternative protection mechanisms with different computational, infrastructure, or hardware-trust requirements [31].

In this study, we use secure multiparty computation (SMPC) because it protects selected client-level intermediate quantities during computation with limited utility trade-offs, while distinguishing between privacy-preserving (PP) computation processes and PP computation results. Traditional FL alone does not guarantee input privacy during aggregation, because intermediate gradients may remain vulnerable to inspection by a curious coordinator [30, 32]. Furthermore, even when inputs are secured, the output privacy of the final global model remains a separate concern, because high-dimensional model parameters or predictions may still leak information about individual participants [29, 31]. Consequently, formal privacy-preserving mechanisms such as SMPC under the standard semi-honest or honest-but-curious assumption are needed to strengthen privacy protection in federated learning for omics data [32–34]. This threat model is particularly relevant to clinical settings, where institutions are generally expected to follow agreed protocols and legal obligations but still require cryptographic protection against inadvertent or curious inspection of sensitive intermediate information [25–27]. SMPC mitigates these threats by cryptographically splitting client updates into secret shares that are aggregated without reconstructing gradients in plaintext at any single party, so intermediate updates are not directly exposed during aggregation under standard SMPC assumptions while collective model improvement remains possible [32, 34]. Recent studies have successfully applied federated learning to downstream scRNA-seq analyses, with some incorporating privacy-enhancing technologies (PETs) [35–38]. In particular, Tabula [39] represents a federated foundation model for scRNA-seq but does not integrate SMPC or comparable PETs for protecting intermediate updates. Consequently, systematic evaluation of privacy-preserving federated fine-tuning and inference for established, benchmarked single-cell foundation models remains limited.

Building on scGPT, a strong candidate for federated foundation modeling in single-cell analysis due to its demonstrated performance across a broad range of scRNA-seq tasks [40], we developed Clifti-GPT, a privacy-preserving federated framework for fine-tuning and applying pretrained foundation models to downstream scRNA-seq analysis. We extend Clifti-GPT beyond prior work in two key ways. First, we enable secure multiparty computation (SMPC)–protected federated fine-tuning and application of foundation models across decentralized clinical cohorts. Second, we introduce transferable inference, in which complementary statistics derived from zero-shot embeddings, such as neighborhood relations or similarity scores, are securely exchanged instead of raw data, embeddings, or local models. This design acknowledges that while fine-tuning remains a privacy-aware endeavor regarding the final model weights, transferable inference prioritizes output privacy. By restricting cross-site exchange to low-dimensional, discrete voting statistics rather than high-dimensional gradients, the framework inherently minimizes the information entropy of the results, making it more resistant to reconstruction attacks. This design allows institutions to benefit from cross-site knowledge for tasks such as reference mapping and annotation transfer while adhering to institutional governance constraints and privacy requirements. We use Crypten [41] for secret-sharing and secure aggregation of both local model updates and locally inferred statistics, aligning the framework with GDPR principles and institutional data-governance practices by design. By leveraging Crypten for the secure aggregation of both local model updates and inferred statistics, Clifti-GPT ensures that the entire computation process is privacy-preserving, as no raw or intermediate data is revealed to any party throughout the fine-tuning or inference workflows. In a comprehensive empirical evaluation, Clift-GPT achieves performance comparable to centralized training for cell type classification while demonstrating rapid convergence in terms of communication rounds across pathologically heterogeneous and imbalanced cohorts. To support consistent, privacy-aware preprocessing, we further devise a privacy-preserving federated binning pipeline that harmonizes count discretization across sites with secure aggregation without centralizing raw data. In addition, our transferable inference pipeline includes an SMPC-protected k-nearest neighbor module for zero-shot reference mapping, complemented by a secure ensemble voting mechanism, enabling collaborative inference with performance comparable to centralized baselines across heterogeneous cohorts. More broadly, integrating foundation models with federated learning offers practical advantages, including expanding data availability without centralized pooling, sharing computational burden across stakeholders, avoiding single-vendor monopolies, and enabling robust multi-task and multi-modality workflows [42]. To our knowledge, Clifti-GPT represents one of the first privacy-preserving federated frameworks for scRNA-seq foundation models, and its design can, in principle, be extended to other benchmarked foundation models and downstream analytical tasks.

## Methods

Leveraging recent advances in foundation models (FMs) for scRNA-seq data, which have demonstrated strong generalization capabilities across diverse datasets, we developed Clifti-GPT, built upon scGPT FM, as a privacy-preserving federated workflow that enables multiple institutions to collaboratively adapt a pretrained FM without sharing raw data. Our approach aims to unlock the full potential of FMs in clinical and multi-institutional settings that are otherwise constrained by privacy, governance, and heterogeneity challenges inherent to biomedical data. The framework supports both fine-tuning for downstream analysis and zero-shot inference, accompanied by complementary distance-based similarity inference. To support privacy preservation, Clifti-GPT integrates additive secret sharing as the core cryptographic mechanism within a secure multi-party computation (SMPC) protocol, such that model updates, label mappings, and other sensitive information are not exposed in plaintext during aggregation and computation. Throughout this work, we adopt the standard semi-honest threat model commonly assumed in SMPC-based federated learning, and rely on established results from prior cryptographic studies demonstrating that additive secret sharing protects intermediate values from direct inspection under these assumptions. This design supports scalability, accommodates heterogeneous data distributions, and provides a foundation for integrating future foundation models with improved embedding architectures in secure multi-party environments.

### Experimental settings

We conducted three categories of experiments to evaluate the effectiveness of our privacy-preserving federated workflow for the scGPT model on downstream tasks. First, we implemented a centralized scenario, in which all client data was aggregated into a single repository and used to fine-tune the pretrained foundation model. This setting serves as an upper-bound performance reference and does not address privacy considerations. Second, we performed local fine-tuning, where each client fine-tuned the pretrained foundation model *F* on its own dataset without sharing any information. While this setting preserves data locality, it may suffer from reduced generalization due to limited local sample sizes. Finally, we developed a federated scenario, where data remain fully decentralized and only model updates and locally inferred statistics are exchanged and aggregated across clients. This setting is designed to align with privacy-preserving and governance-aware data-analysis requirements while overcoming data-sharing restrictions across institutions. Crucially, the federated experiments are controlled simulations of multi-client deployment rather than deployed institutional trials. To ensure reproducibility and enable direct comparison among centralized, local, and federated baselines, we used public datasets and defined clients from real dataset annotations, including cohort, study, disease status, brain region, batch, and patient/sample origin where available. This design captures substantial biological and technical non-IID heterogeneity while retaining the controls needed to isolate the effects of federated optimization and SMPC-protected aggregation.

### Evaluation protocol

To ensure comparability across scenarios, we trained all models on the entire reference set *R* and evaluated performance using a held-out query set *Q*. Gene tokens used for fine-tuning were aligned with those in the pretrained foundation model, and all gene expression values were normalized consistently across all settings. In the federated scenario, the common set of genes across clients was aggregated using a secure hashing mechanism, thereby concealing the presence or absence of specific genes at each site. For cell type annotation tasks, gene expression values were log-transformed and binned prior to fine-tuning. We assume *C* clients *1, … ,C*, each client *i* with ${n_i}$ training samples, collaboratively fine-tune or apply the scGPT foundation model *F* on their local data without ever exchanging raw data. Clients communicate their local updates using additive secret sharing ${\left[\kern-0.15em\left[ \cdot \right]\kern-0.15em\right]_P}$ among *P* computational parties. Under this scheme, sensitive quantities remain secret-shared $\langle \cdot \rangle$ throughout computation, and only aggregated values are revealed in plaintext during the final aggregation step.

## Datasets

In this study, we applied consistent data preparation procedures for both reference mapping and cell type classification tasks. For each dataset, cells were partitioned into reference and query sets, ensuring that all cell types present in the query set were also represented in the reference set. Reference cells were assigned to clients based on batch origin to simulate realistic federated learning scenarios encompassing diverse technical, biological, and disease-driven heterogeneity. Consistent with established protocols, we utilized the pre-selected highly variable genes (HVGs) from the scGPT study [5], identified across the reference cohorts through a label-agnostic selection process.

The Multiple Sclerosis (MS) [43] dataset, comprising 21,312 cells across 18 distinct cell types, was collected from three brain regions (prefrontal cortex, cerebral cortex, and premotor cortex) in both healthy controls and MS patients. To simulate a realistic federated learning scenario with biological and disease-driven heterogeneity, we defined four reference clients: control-prefrontal, MS-prefrontal, control-cerebral, and MS-cerebral, encompassing a total of 18,739 cells. The remaining 2,573 cells, derived from the control and MS premotor cortex, were designated as the query dataset (See Additional file 1: Table 1). For downstream analysis, we selected 3,000 highly variable genes (HVGs).

The Covid-19 [44] dataset includes 19,922 cells spanning 36 immune and epithelial cell types, collected from nine distinct cohorts: 10X, Covid, HCL, Northwestern, Oetjen, Sanger, Freytag, Krasnow, and Sun. To simulate a realistic federated learning scenario with diverse technical and biological batch effects, we assigned six cohorts (10X, Covid, HCL, Northwestern, Oetjen, Sanger) as reference clients (16,977 cells), and designated three cohorts (Freytag, Krasnow, Sun) as the query dataset (2,945 cells). This split ensures label consistency without requiring any filtering, as all cell types present in the query set were also represented in the reference set. We selected 1200 HVGs.

The Human Pancreas (HP) dataset comprises 14,746 cells spanning 11 cell types, sourced from five studies: Baron [45], Muraro [46], Wang [47], Xin [48], and Segerstolpe [49]. To simulate a federated learning scenario, we designated four studies (Baron, Muraro, Wang, and Xin) as reference clients, contributing a total of 12,684 cells, and assigned Segerstolpe (2,062 cells) as the query dataset. We selected 3,000 highly variable genes (HVGs) for downstream analysis.

The Lung-Kim dataset [50] includes 30,472 cells spanning 10 cell types, collected from 14 primary lung adenocarcinoma patient samples. We simulate a federated scenario with 10 clients (P0006, P0008, P0018, P0020, P0025, P0028, P0030, P0034, P1028, and P1058) as the reference set (23,185 cells) and the rest of samples (P0019, P0031, P1006, P1049) as the query set (7,287 cells). We selected 3,000 highly variable genes (HVGs) for downstream analysis.

The Cell Line (CL) [51] dataset comprises 9,531 cells spanning two cell types (293T and Jurkat) profiled across three batches. To simulate a federated learning scenario, we included two batches as reference clients (2,885 cells), and designated one batch containing a mix of both cell types as the query set (3,388 cells). A total of 1,126 highly variable genes (HVGs) were selected for downstream analysis.

The Myeloid dataset [52] comprises 13,178 cells spanning 16 annotated immune cell types, collected across 84 distinct batches. To simulate a large-scale federated learning scenario with considerable technical and biological variability, we designated 30 batches (10,555 cells) as reference clients and 54 batches (2,623 cells) as the query dataset. For downstream analysis, we selected 3,000 highly variable genes (HVGs). To evaluate scalability, we designed four federated scenarios based on the number of clients involved. To ensure that all scenarios use the same set of training samples, we defined the $\mathit{top-N}$ setting, where $N \in \left\{ {5,10,20,30} \right\}$, by assigning the *N - 1* largest batches (based on cell count) as individual reference clients, and grouping the remaining batches into a single reference client labeled “rest.” In the top-30 scenario, each reference client corresponds to a unique batch, thereby preserving the full diversity of the reference data.    

### Federated binning

Effective federated adaptation of foundation models requires preprocessing pipelines tailored to the architecture of a specific target FM. Within the Clifti-GPT framework, we utilized centralized highly variable gene (HVG) selection; however, the scGPT tokenization strategy employs localized per-cell binning to convert continuous gene expression into discrete representations. This approach may present significant analytical hurdles when applied across pathologically heterogeneous federated cohorts. To address this challenge, we introduced a secure, cross-matrix federated binning protocol (Additional file 2: Algorithm 1) designed to standardize discrete bins across multiple clients for downstream federated analyses. This approach eliminates the need to centralize raw data and ensures that intermediate statistics are not disclosed. To ensure that the coordinator cannot observe any individual quantile vector or specific sample sizes, each client contributes a secret-shared local quantile vector $\langle {B_i}\rangle$ and a non-zero count $\langle {n_i}\rangle$. The coordinator then securely computes ${B_{global}}$ via a sample-size weighted average, followed by a final quantile pass to enforce monotonic spacing.

$$\left\langle {{B_{global}}} \right\rangle \leftarrow \mathop \sum \limits_i \left\langle {{n_i}} \right\rangle \left\langle {{B_i}} \right\rangle \;/\mathop \sum \limits_i \left\langle {{n_i}} \right\rangle $$

To further address possible statistical bias in averaging per-client quantile vectors as compared to a centralized pooled quantile, we implemented a federated secure histogram binning protocol based on SMPC. As detailed in Additional file 2: Algorithm 2, this methodology enables the reconstruction of global quantile cuts from a pooled empirical cumulative distribution while ensuring that individual per-client histograms remain private. The framework employs a federated secure histogram binning protocol to facilitate the secure computation of global quantile cuts (${B_{global}}$) while ensuring that individual client data remains confidential. During the initial communication phase, participating clients execute a secret-shared maximum computation to determine the global maximum non-zero expression $max\_expr$, which defines a shared public grid *g* consisting of *M + 1* edges. In the subsequent round, local histograms ${H_i}$ and non-zero counts ${n_i}$ are secret-shared by each client, enabling the coordinator to perform secure aggregation under SMPC to obtain secret-shared global sums $\langle H\rangle$ and $\langle N\rangle$. The quantile-cut routine is then carried out in the SMPC domain by computing secret-shared cumulative histogram entries $\langle {S_m}\rangle$ for *m = 1, … ,M* and applying secure comparison masks to identify the target bin boundaries of the pooled empirical cumulative distribution. The final global bin-edge vector ${B_{global}}$ is computed under secret sharing and revealed only at the end of the routine.

### Privacy-preserving federated fine-tuning

In Clifti-GPT, the coordinator initializes the pretrained foundation model weights and distributes the initial model ${W_0}$ to all participating clients. Each client fine-tunes the model locally following the original scGPT [5] training protocol, including architecture, optimization strategy, and hyperparameter settings. To enable privacy-preserving federated training in a semi-honest setting, clients do not share raw data or plaintext model updates. Instead, each client secret-shares its local model updates ${\left[\kern-0.15em\left[ {{W_i}} \right]\kern-0.15em\right]_P}$ using additive secret sharing within a SMPC protocol, such that intermediate parameters are not exposed in plaintext to any single party during aggregation. For model aggregation, we employed FedAvg, where the coordinator computes the weighted mean of local model parameters:

$${W_{t + 1}} = {{\mathop \sum \nolimits_{i = 1}^C {n_i} \times {W_i}} \over N}$$

where $N = \mathop \sum \limits_{i = 1}^C {n_i}$ is the total number of samples across all clients, and ${W_t}$ denotes the global model parameters at round *t*. This weighting gives larger datasets proportionally greater influence on the global model. We also experimented with FedProx to address data heterogeneity and improve convergence. In this approach, a proximal term is added to the local objective:

$${\mu \over 2}\parallel{W_i} - {W_t}\parallel{^2}$$

where *μ * is the proximal coefficient that constrains local updates to remain closer to the global model. Aggregation then follows the same weighted averaging as FedAvg but benefits from improved stability when client data distributions differ. The aggregation process is repeated over multiple communication rounds until the global model converges. Using secure aggregation based on SMPC, Clifti-GPT enables privacy-preserving collaborative fine-tuning of foundation models across multi-institutional scRNA-seq datasets.

### Privacy-preserving transferable inference for zero-shot FM applications

Zero-shot applications of foundation models, such as reference mapping for cell type annotation, typically rely on comparing query embeddings against reference embeddings using distance-based similarity measures. In privacy-sensitive and federated settings, however, directly sharing embeddings or local model representations across institutions can leak sensitive information and is therefore not permissible. To address this limitation, we introduce transferable inference, a privacy-preserving inference paradigm for zero-shot applications in which predictions are performed collaboratively across decentralized sites without transferring raw data, embeddings, or local models. Instead, only complementary statistics derived from comparisons of secret-shared embeddings, such as nearest-neighbor distances and votes, are exchanged and aggregated under SMPC. This enables transferable inference, whereby inference-relevant information is transferred across clinical cohorts without exposing sensitive data, in accordance with privacy and governance requirements.

As an instantiation of transferable inference, we developed a privacy-preserving federated workflow for zero-shot reference mapping (Additional file 2: Algorithm 3), where all parties agree on a shared embedding model *F*, and compute:

$$Q{^{\prime}} = F\left( Q \right),\quad {R_i}^\prime = F\left( {{R_i}} \right)\quad \left( {i = 1, \ldots ,C} \right).$$

Then clients calculate local distance matrices ${D_i}$ and index matrices ${I_i}$ via $\left( {{D_i},{I_i}} \right) = \parallel{Q{^{\prime}} - {R_i}^\prime}\parallel$ and retaining only $\mathit{top-k}$ neighbor entries. The server collects these local $\mathit{top-k}$ index lists, merges them to form a global neighbor set for each query, and redistributes this global index list back to the clients. Finally, each client casts votes, by looking up its own cell-type labels for the selected neighbors, and returns the vote counts ${V_i}$ to the server. Consequently, the server aggregates client votes ${V_{sum}} = \mathop \sum \limits_{i = 1}^C {V_i}$ to select the most frequent label as the final prediction for each query cell. This protocol is designed such that raw reference data remains local to each client while still enabling accurate zero-shot nearest-neighbor classification.

To support privacy-preserving transferable inference under a semi-honest threat model, we customize the transferable inference pipeline for reference mapping by applying additive secret sharing to both query and reference statistics, enabling local and global KNN searches followed by secure voting (Additional file 2: Algorithm 4). Importantly, we refrain from disclosing these statistics in cleartext; instead, they remain secret-shared and are only aggregated under SMPC, such that raw data and intermediate quantities are not exposed in plaintext outside the originating institution. First, we secret-share the query embeddings *Q\prime* among *P* parties $\langle Q\rangle = {[[Q{^{\prime}}]]_P}$. Then the coordinator collects client’s plaintext labels ${l_i}$ into the global label set *L* and define a bijection $\Phi :L \to \left\{ {1, \ldots ,\left| L \right|} \right\}$ that assigns each unique label string an integer index, assuming the labels are harmonized across clients. Next, the coordinator securely aggregate the clients’ local sample counts ${n_i}$ to compute the global offset *O*, thereby defining disjoint index ranges for each client. Accordingly, clients map their cell-type labels to global indices $l \leftarrow \left[ {\Phi \left( {{l_{i,r}}} \right)} \right]_{r = 0}^{\left| {{R_i}} \right| - 1}$ and secret-share $\langle {l_i}\rangle$ besides their embedded reference data $\langle {R_i}\rangle$ and local offset index vector $\langle {r_i}\rangle$ (Additional file 2: Algorithm 5). Each client then triggers the secret-shared squared Euclidean distance matrix calculation between reference cells and secret-shared target query cells:

$$\langle D_i^{\left( 0 \right)}\rangle = \left( {\langle Q\rangle \circ \langle Q\rangle } \right){\mkern 1mu} 1_{\left| {{R_i}} \right|}^T + {1_{\left| Q \right|}}{\mkern 1mu} {\left( {\langle {R_i}\rangle \circ \langle {R_i}\rangle } \right)^T} - 2{\mkern 1mu} \langle Q\rangle {\mkern 1mu} {\langle {R_i}\rangle ^T},$$

where * ∘ * denotes element-wise multiplication and ${1_n}$ is an $\mathit{n-vector}$ of ones. Afterwards, each client secretly selects its local *k* nearest neighbors for each query, producing per-client $\it{top-k}$ distance and index shares:

$$\langle {T_i}\rangle = \left[ {{\mkern 1mu} 1_{\left| {{R_i}} \right|}^T\left( {minMask\left( {\langle D_i^{\left( {j - 1} \right)}\rangle } \right) \odot \langle D_i^{\left( 0 \right)}\rangle } \right)} \right]_{j = 1}^k.$$

$$\langle {I_i}\rangle = \left[ {{\mkern 1mu} minMask\left( {\langle D_i^{\left( {j - 1} \right)}\rangle } \right){\mkern 1mu} \langle {r_i}\rangle } \right]_{j = 1}^k.$$

Here, *minMask* performs a secret-shared *argmin* in one-hot form to locate the nearest-neighbor entries (Additional file 2: Algorithm 6). Finally, computational parties concatenate all clients’ $\it{top-k}$ shares along the neighbor axis to form the global secret-shared distance matrices:

$$\langle D\rangle = \left[ {{\mkern 1mu} \langle {T_1}\rangle ,{\mkern 1mu} \langle {T_2}\rangle ,\> \ldots ,{\mkern 1mu} \langle {T_C}\rangle } \right]\> \in \>{F^{\,\left| Q \right| \times \left( {C\,k} \right)}},$$

and likewise for the indices $\langle I\rangle$. Next, the coordinator locates the global *k* nearest neighbors by iteratively applying the one-hot *argmin* to the concatenated distance matrix:

$$\langle N\rangle = \left[ {{\mkern 1mu} minMask\left( {\langle {D^{\left( {j - 1} \right)}}\rangle } \right){\mkern 1mu} {{\langle I\rangle }_{:,j}}} \right]_{j = 1}^k{\mkern 1mu} ,$$

where, after each selection, we suppress the selected minima using a large constant *Λ *:

$$\langle {D^{\left( j \right)}}\rangle = \langle {D^{\left( {j - 1} \right)}}\rangle + {\rm{\Lambda }}{\mkern 1mu} minMask\left( {\langle {D^{\left( {j - 1} \right)}}\rangle } \right),$$

so that $\langle {D^{\left( 0 \right)}}\rangle$ is the fully concatenated distance matrix (Additional file 2: Algorithm 6).

In the voting phase (Additional file 2: Algorithm 7), each client matches global neighbor indices to its own reference indices to cast its vote:

$$\left\langle {{V_i}} \right\rangle = \mathop \sum \limits_{j = 1}^k oneHot\left( {eqMask\left( {{{\left\langle N \right\rangle }_{:,j}},\,\left\langle {{r_i}} \right\rangle } \right)\,{{\left\langle l \right\rangle }_i},\;{n_l}} \right),$$

where, $eqMask\left( {{{\langle N\rangle }_{:,j}},{\mkern 1mu} \langle {r_i}\rangle } \right)$ produces a one-hot vector marking exactly those positions where the *j*th global neighbor index matches client *i*‘s local reference index. Multiplying this mask by $\langle {l_i}\rangle$ yields the scalar vote $\langle v_i^{\left( j \right)}\rangle$ which is then expanded to a length of ${n_l}$ one-hot vector (all zeros if *v = 0*). Next, clients accumulate their $\mathit{top-k}$ votes into $\langle {V_i}\rangle$, and the coordinator securely produces the global vote matrix $\langle V\rangle$. All operations triggered by either clients or coordinators are conducted in secret shared format across computational parties. Finally, the coordinator extracts the most frequent vote for each query by applying a one-hot maximum mask on the secret-shared global votes matrix, revealing only the final prediction:

$${M^{plain}} = { \llparenthesis{maxMask(\langle V\rangle })\rrparenthesis}_P,$$

where $maxMask\left( {\langle V\rangle } \right)$ produces a one-hot encoding of the index with the maximum vote count for each query, and $\llparenthesis\cdot\rrparenthesis$ denotes the SMPC aggregation of secret-shares from *P* parties being revealed to the coordinator. The results are then mapped to labels using the global label-index vector *ϕ *, yielding the federated reference predictions ${M^{plain}}\,\phi.$  

Through this procedure, inference-relevant information is aggregated across institutions without exposing raw embeddings, reference identities, or intermediate statistics, realizing transferable inference for zero-shot reference mapping under privacy-preserving federated settings.

We further evaluated the computational overhead of privacy-preserving federated Clifti-GPT workflows by comparing standard plaintext execution with CrypTen-based SMPC across three core procedures: federated fine-tuning aggregation, federated reference mapping, and federated binning. Runtime was measured across representative client counts, model sizes, query and reference sizes, neighborhood sizes, and binning strategies using repeated GPU-based experiments (Additional file 2: Section 3).

### Batch effect correction

To study the impact of batch effects on downstream performance, we employed scGen [53] for batch effect removal, leveraging its variational autoencoder (VAE) framework to learn a latent representation that disentangles biological signals from technical variation. scGen optimizes a combined reconstruction and Kullback–Leibler (KL) divergence loss:

$$L\left( {{x_i}} \right) = {E_{z \sim Q\left( {z|{x_i};\phi } \right)}}\left[ {log P \left( {{x_i}|z;\theta } \right)} \right] + KL\left[ {Q\left( {z|{x_i};\phi } \right)\,\parallel\,P\left( {z|{x_i};\theta } \right)} \right]$$

Following training, mean latent features for each shared cell type are computed in a reference batch, and the latent representations of other batches are shifted toward these means. This correction procedure aligns cell-type-specific embeddings across batches, aiming for mitigating technical variability and preserving biological heterogeneity. Batch effect correction was applied solely for analysis purposes to assess its influence on centralized and federated performance and was not integrated into the Clifti-GPT workflow.

## Results

**Fig. 1:**
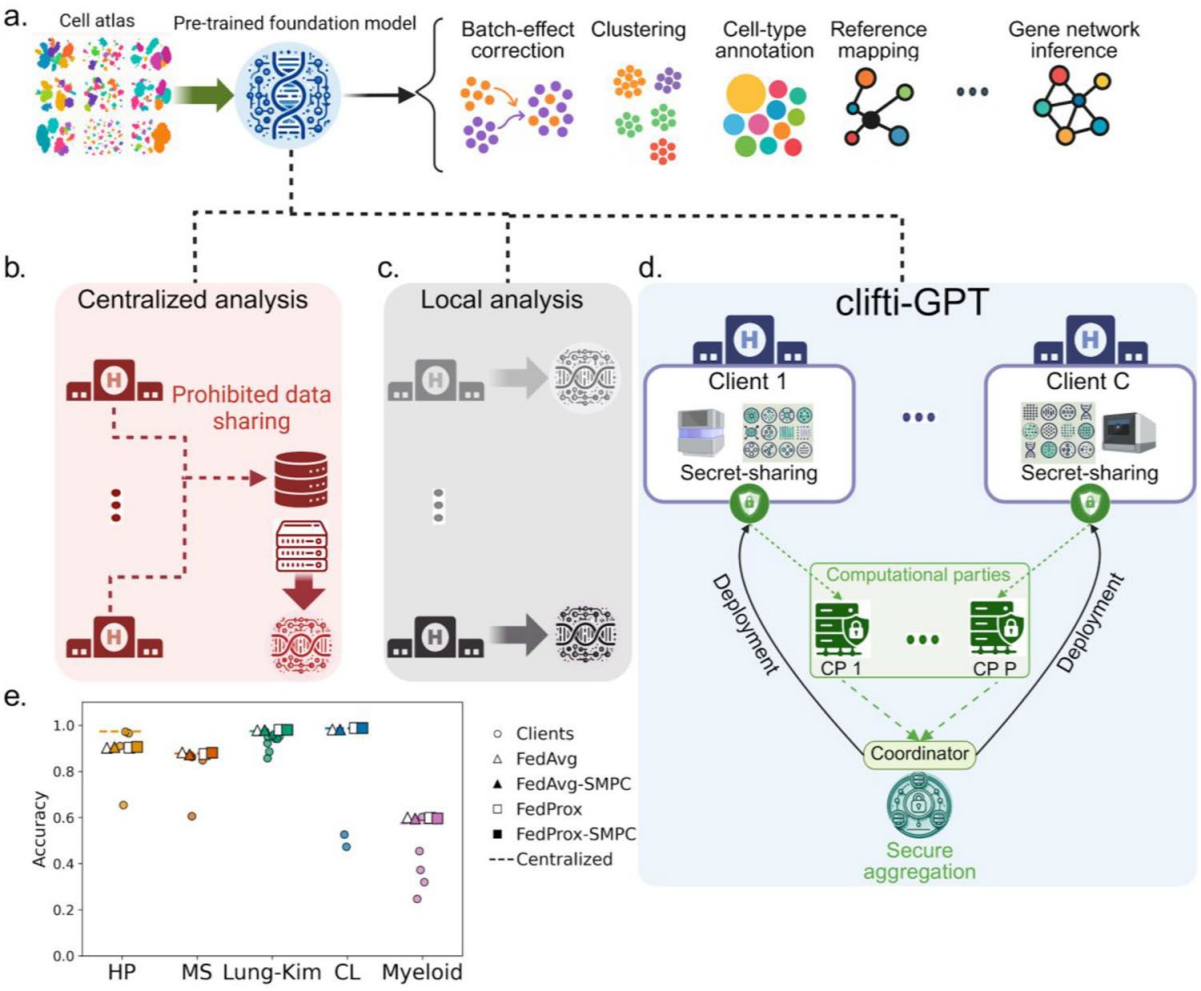
Overview of the Clifti-GPT privacy-preserving federated training and evaluation workflow. (**a**) Pre-trained scGPT on a comprehensive cell atlas serves as a versatile backbone but requires downstream fine-tuning to achieve optimal task-specific performance. (**b**) Centralized fine-tuning of scGPT risks violating patient privacy and institutional data-governance policies. (**c**) In the absence of data sharing, hospitals independently fine-tune the pre-trained model on their limited local cohorts, often resulting in suboptimal performance. (**d**) In our privacy-preserving federated workflow, each hospital trains locally and splits its model updates into cryptographic secret shares, which are distributed to independent computational parties. These parties aggregate the shares using secure multiparty computation (SMPC) without reconstructing any client’s gradients, producing a global model that is redistributed for the next training round. (**e**) The federated models achieve accuracy comparable to centralized fine-tuning and outperform local models in cell type classification across the MS, Lung-Kim, CL, and Myeloid datasets, while showing competitive performance on HP under the default training budget

We introduce Clifti-GPT, a privacy-preserving federated framework for fine-tuning and applying the scGPT foundation model to downstream scRNA-seq analyses. Pre-trained on cell atlas, with over 33 million cells [10], to capture the full complexity of single-cell data (Fig. 1a), scGPT delivers high performance but typically requires fine-tuning on large, heterogeneous cohorts that are often siloed across hospitals and cannot be centrally pooled for privacy or governance reasons. Although data sharing could potentially yield the most accurate results, it is frequently prohibited (Fig. 1b). Consequently, local clients are restricted on training on individual datasets as an alternative (Fig. 1c). To address these challenges and enable collaborative analysis under privacy and governance constraints, Clifti-GPT distributes a pretrained scGPT model to participating sites, where local fine-tuning is performed and model updates are aggregated using SMPC-based secure aggregation (Fig. 1d). The resulting global model is iteratively refined across communication rounds. To assess the effect of secret-sharing on performance, we also implemented a non-SMPC variant. Across all the experiments we used federated binning without SMPC.

We refer to our federated models by the aggregation algorithm used (FedAvg [20] or FedProx [54]), each of which can be accompanied by SMPC. All experiments (local, centralized, and federated) used the same scGPT architecture and hyperparameter settings, including a batch size of 32 and the default optimization configuration described in the original scGPT study. Centralized and local models were trained for 20 epochs, following the standard scGPT training protocol for cell type classification across all evaluated datasets. To provide a simple and consistent reference for federated evaluation without hyperparameter tuning, federated models were trained using a fixed budget of one local epoch per client over 20 communication rounds (Fig. 1e); later, we explore the effect of alternative federated training budgets. We evaluated cell type classification performance based on accuracy, precision, recall, and Macro-F1 across five single-cell RNA-seq datasets with realistic federated scenarios: four clients for the Multiple Sclerosis (MS) [43], four for Human Pancreas (HP) [45–49], two for Cell Line (CL) [51], ten for Lung-Kim [50], and five for the Myeloid [52] (Additional File 1: Tables 1–6). For the Myeloid dataset, we assigned the four largest batches (by cell count) to four individual clients and grouped the remaining batches into a fifth reference client. In all other datasets, each client corresponded to a unique batch, reflecting biological, technical, and disease-driven heterogeneity (Additional File 1: Tables 1–6, Additional file 3: Figures S1-S2).

### Clifti-GPT enables effective, privacy-preserving federated cell-type annotation

While centralized annotation yields the highest accuracy, it is often prohibited or avoided due to privacy and data governance concerns. Under privacy constraints, local training on the limited private data available at hospitals, though feasible, underperforms the centralized training setting in which all data are accessible (Fig. 1e). Clifti-GPT, trained for one local epoch and 20 communication rounds, achieves accuracy comparable to centralized scGPT while outperforming local client models across the MS, Lung-Kim, CL, and Myeloid datasets. Similar performance improvements are observed for CL and Lung-Kim datasets across precision, recall, and macro-F1, while other datasets exhibit slight drops in some metrics (Additional file 3: Fig. S3). We explored different numbers of local epochs and communication rounds for FedAvg with and without SMPC (Additional file 3: Fig. S4), showing that FedAvg-SMPC performance improves with increased local training and communication. This compensates for the shortcomings observed in the one-epoch, 20-round setting for precision, recall, and F1 scores on the MS and Myeloid datasets. Similarly, we evaluated the impact of varying hyperparameters, including the proximal term $\mu \in \left\{ {0.01,0.05,0.1,0.2,0.5} \right\}$ for FedProx-SMPC aggregation (Additional file 3: Fig. S5). We observed that increasing *μ * generally requires more communication rounds to achieve convergence. In contrast, FedProx-SMPC with *μ = 0.01* performs notably better than FedAvg-SMPC across most cases requiring fewer communication rounds (Additional file 3: Fig. S5). For the HP dataset, 20 communication rounds appear insufficient to fully match centralized accuracy, although FedAvg-SMPC shows competitive performance in precision, recall, and macro-F1 (Additional file 3: Fig. S3). By increasing the number of communication rounds to 156, FedAvg-SMPC reaches within 1% of the centralized model’s accuracy.

### Clifti-GPT demonstrates strong communication efficiency in federated cell type annotation

**Fig. 2:**
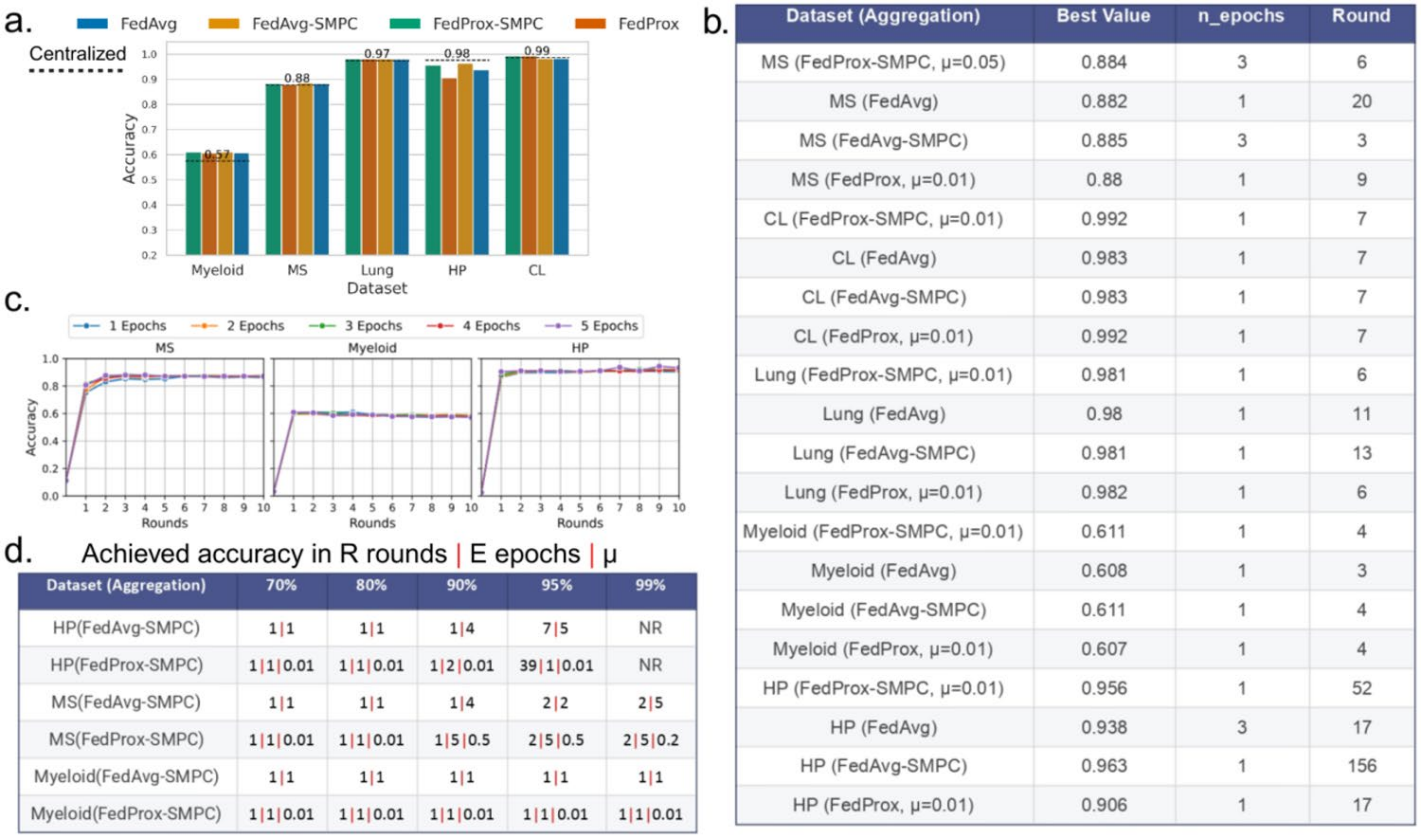
Hyperparameter tuning enhances the robustness and communication efficiency of Clifti-GPT in cell type classification across HP, CL, Myeloid, MS, and Lung datasets. (**a**) Best accuracy achieved using FedAvg and FedProx aggregation strategies, with and without SMPC, compared to centralized scGPT performance. (**b**) Corresponding training configurations (number of communication rounds, local training epochs, and proximal term *μ *) for each result shown in panel a, highlighting the diversity of optimal settings across datasets and aggregation strategies. (**c**) Increasing the number of local training epochs in FedAvg-SMPC on MS, Myeloid, and HP datasets does not yield accuracy improvements, indicating limited benefit from additional client-side computation. (**d**) Using FedAvg-SMPC and FedProx-SMPC, different margins (70% to 99%) of centralized accuracy can be reached under varying communication budgets, demonstrating flexible communication efficiency. Each cell displays the number of communication rounds, local epochs, and µ value (only applicable for FedProx), separated by the symbol |, while “NR” indicates that the specified accuracy threshold was not reached within the evaluated training budget

Across the CL, MS, Lung, and Myeloid datasets, the best-performing aggregation strategies match the accuracy of the centralized scGPT model on corresponding datasets (Fig. 2a). A similar trend holds for precision across datasets (Additional file 3: Fig. S6). However, using SMPC slightly reduces recall on the MS dataset and macro-F1 scores on both the MS and Myeloid datasets. In general, SMPC introduces a modest performance drop for both FedAvg and FedProx, with the exception of FedProx-SMPC precision, which remains nearly identical to its non-SMPC counterpart (Additional file 3: Fig. S6b). The results in Fig. 2a are achieved using varying hyperparameter settings, as detailed in Fig. 2b. Notably, one local training epoch consistently yields the highest accuracy across the evaluated datasets. This pattern is consistent for other metrics as well (Additional file 3: Fig. S7). To assess the impact of increased local computation, we examined the effect of increasing the number of local epochs in FedAvg-SMPC across the MS, Myeloid, and HP datasets. As shown in Fig. 2c, additional local computation does not lead to notable improvements in accuracy. We investigated the communication efficiency by measuring rounds required to achieve accuracy thresholds (70–99%) of centralized performance across the HP, MS, and Myeloid datasets. As shown in Fig. 2d, both FedAvg-SMPC and FedProx-SMPC achieve 90% of centralized accuracy using just a single communication round and varying number of local epochs. Furthermore, 99% of centralized accuracy on MS and Myeloid is reached in as few as two rounds, demonstrating high communication efficiency on these datasets. While neither FedAvg-SMPC nor FedProx-SMPC on the HP dataset reach 99% of centralized accuracy within the evaluated training budget, both strategies achieve 99% of the centralized model’s performance on other metrics (precision, recall, macro-F1) in fewer than four rounds (Additional file 3: Fig. S8). Although the federated model trails the centralized model in accuracy on the HP dataset, the difference is minimal at 0.01 (Additional file 3: Fig. S9).

### Clifti-GPT demonstrates competitive classification performance across various cell types compared to centralized models

**Fig. 3:**
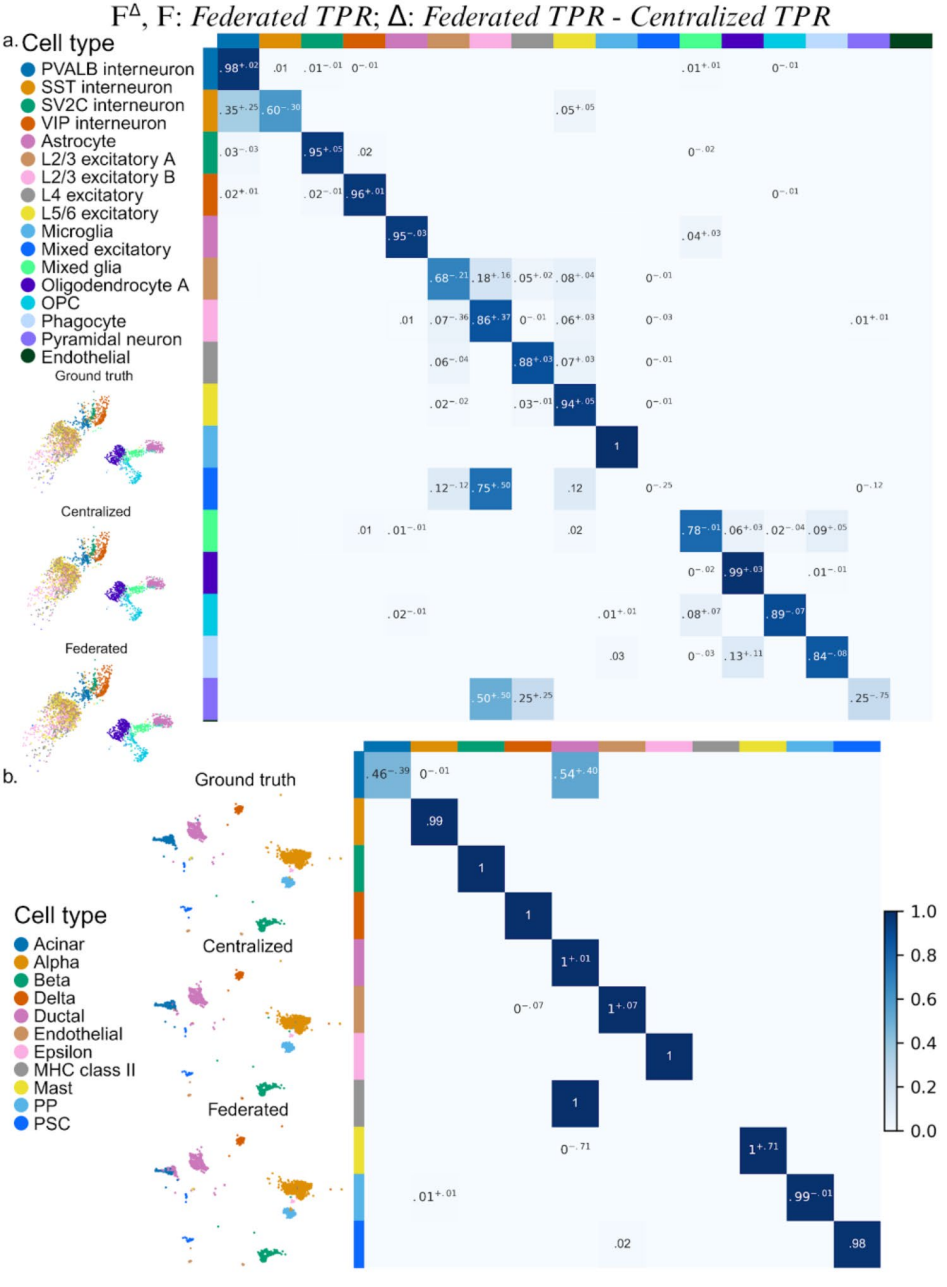
Confusion matrices (per-class TPRs) and UMAP visualization of the best federated models compared with centralized training, demonstrating comparable performance across various cell types. Each cell shows $F^\Delta$ where *F* is federated TPR and *Δ* shows the TPR difference between federated and centralized models. (**a**) On the MS dataset, using Fed Prox-SMPC (*μ = 0.01*), trained for two epochs and four rounds, the model closely matched centralized per-class TPRs for abundant cell types, with minor decrease for Phagocytes as a low-represented cell type. (**b**) On the HP dataset, Fed Avg-SMPC, trained for three epochs and 21 rounds, preserved or improved centralized per-class TPRs for common cell types; among low-represented cell types, it achieved a large gain for Mast cells and matched the centralized model on Epsilon cells

We analyzed the per-class true positive rate (TPR) of the best federated model against centralized training, considering the low-represented cell types in the reference data. TPRs of the federated model are shown using $TP{R^\Delta }$, where $\Delta = TP{R_{fed}} - TP{R_{Cent}}$ denotes the difference between federated and centralized TPR, and a positive value indicates improved TPR in the federated model. Using FedProx-SMPC, trained for two epochs and four rounds with *μ = 0.01*, the model achieved an overall recall of 0.79 on MS dataset, closely matching the centralized recall of 0.81 (Fig. 3a). In general, per-class TPRs were well preserved, with slight improvements for SV2C interneurons ${.95^{ + .05}}$ and PVALB interneurons ${.98^{ + .02}}$. For Phagocytes as a low-represented cell type with 47 cells in the reference data, the TPR is ${.84^{ - .08}}$, indicating a minor decrease compared to centralized training (Fig. 3a, Additional file 1: Table 1).

Using FedAvg-SMPC, trained for three epochs and 21 rounds, the model achieved an overall recall of 0.86 on the HP dataset. In general, per-class TPRs were preserved or improved for common cell types, e.g., Alpha, Beta, Delta, Ductal, and Endothelial (Fig. 3b). Among rare cell types, the federated model showed a notable gain with 0.71 TPR in Mast cells (25 cells) and matched the perfect TPR of the centralized model on Epsilon cells (21 cells). However, both the centralized and federated models misclassified the single MHC class II cell (1 cell in reference) as a Ductal cell, while FedAvg-SMPC shows notable −0.39 TPR loss on Acinar cells (Fig. 3b, Additional file 1: Table 2).

For the Lung-Kim dataset, FedAvg-SMPC with one epoch and seven rounds reached a recall of 0.98, with notable gains for certain classes such as Malignant (+0.02) and NK_cell (+0.01) (Additional file 3: Fig. S10a). This strong alignment was observed despite imbalanced cell type distributions across batches (Additional file 1: Table 3). For the Dendritic cell type, which had only 87 cells in the reference set, the federated model achieved a recall gain of 0.07. Similarly strong performance was observed on the CL dataset, where FedProx-SMPC (*μ = 0.01*), trained for one epoch and seven rounds, achieved a recall of 0.99, identical to the centralized model, with a + 0.01 recall gain for 293t and matching recall for jurkat (Additional file 3: Fig. S10b; Additional file 1: Table 4).

### Privacy-preserving federated reference mapping achieves performance comparable to or exceeding the centralized baseline on the HP, CL, Lung-Kim, and MS datasets

**Fig. 4:**
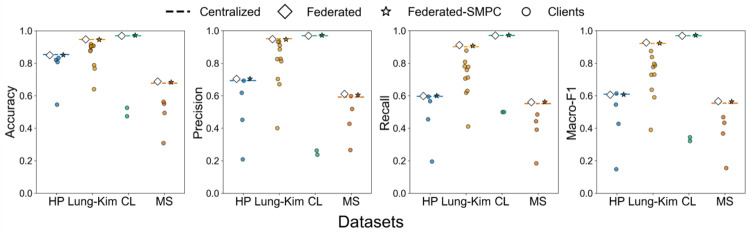
Privacy-preserving federated reference mapping on HP, Lung-Kim, CL, and MS datasets improves client performance and matches the centralized model across all metrics. From left to right, panels show accuracy, precision, recall, and macro-F1. The horizontal line represents centralized reference mapping performance. Diamonds and stars represent the federated model without and with SMPC, respectively. Circles depict the performance of each client’s local reference mapping using its limited private data.

Beyond fine-tuning, Clifti-GPT also enables transferable inference in zero-shot applications such as reference mapping, where only complementary statistics, e.g., distances or neighborhood relations, are exchanged instead of raw data or embeddings. In this setting, foundation models provide the embeddings, while transferable inference supplies the necessary computation; for example, reference mapping assigns query cells based on the majority of their closest reference neighbors. We evaluated privacy-preserving federated transferable inference for zero-shot reference mapping, with and without SMPC, against the centralized model using accuracy, precision, recall, and macro-F1 (Fig. 4). The federated SMPC model matches the centralized performance on the CL dataset across all metrics, matches or improves all metrics for HP and Lung-Kim, and outperforms the centralized model by a small margin (+0.01) across all metrics for MS dataset (Additional file 3: Fig. S11).

### Privacy-preserving federated models demonstrate scalability on the Myeloid dataset distributed across different numbers of clients

**Fig. 5:**
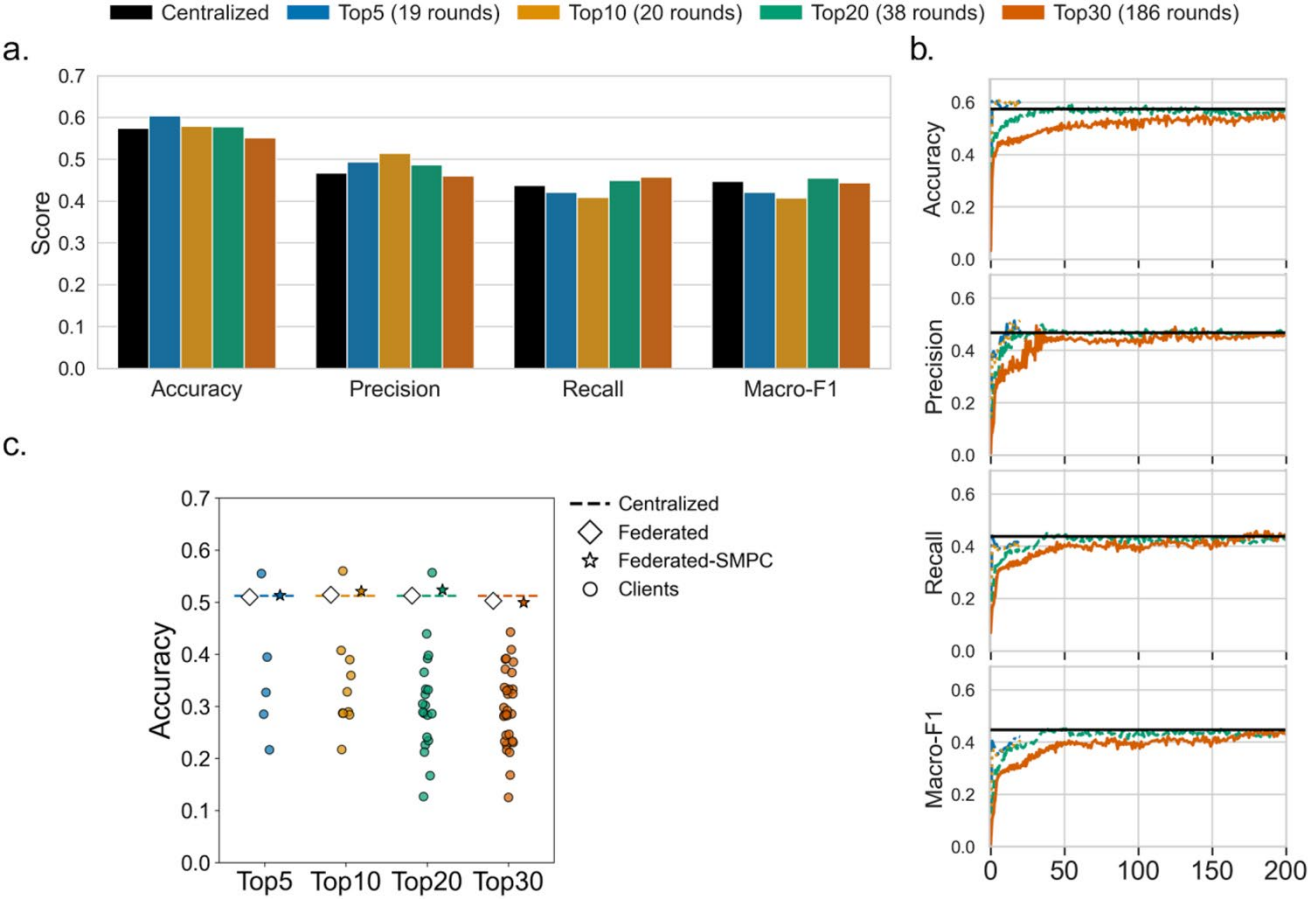
Scalability of privacy-preserving federated annotation and reference mapping on the distributed Myeloid dataset across 30 clients, demonstrating performance comparable to centralized models and superior to local training. (**a**) Cell type annotation performance of FedProx-SMPC trained for one local epoch and an optimal number of communication rounds (shown in the legend) across different scalability scenarios of Myeloid dataset with 5, 10, 20, and 30 clients (Top5, Top10, Top20, and Top30). (**b**) convergence curves of FedProx-SMPC for cell type classification over 200 communication rounds across different scalability scenarios. (**c**) reference mapping accuracy of federated models with and without SMPC across different scalability scenarios

To assess federated learning scalability, we distributed the Myeloid dataset across varying numbers of clients to create scenarios with increasing data heterogeneity. Accordingly, we sorted the Myeloid reference batches by cell count and partitioned them into 5, 10, 20, and 30 clients, corresponding to four federated scenarios: Top5, Top10, Top20, and Top30. Each client was assigned a dedicated batch across all scenarios, except for the “rest” client in the Top5, Top10, and Top20 (Top5-20) scenarios, which received the remaining batches from the reference data. This setup produced scalable scenarios with varying degrees of cell-type heterogeneity across clients (Additional file 3: Fig. S3). We compared the performance of FedProx-SMPC (*μ = 0.01*), trained for one epoch, with the centralized model across scalability scenarios (Fig. 5a). FedProx-SMPC achieved its best accuracy after 19 rounds for Top5, 20 rounds for Top10, 38 rounds for Top20, and 186 rounds for Top30, matching the centralized baseline across scalability settings. The “rest” client in Top5–Top20 outperformed both centralized and federated models in accuracy and recall, but in the realistic Top30 scenario lacked sufficient data (Additional file 3: Fig. S12). While some configurations, such as Top5, showed lower recall and macro-F1, these limitations could be mitigated by adjusting the number of rounds, training epochs, and *μ * (Additional file 3: Fig. S9). For example, FedAvg-SMPC trained on Top5 for three epochs and 17 rounds matched the recall and macro-F1 of the centralized baseline (Additional file 3: Fig. S9). The effect of extended training over 200 communication rounds was also examined (Fig. 5b), showing steady accuracy improvements for Top20 and Top30. In the most heterogeneous Top30 case, substantially more communication rounds were required for convergence, whereas Top5 and Top10 converged in fewer rounds.

We specifically examined federated performance across various cell types under increasing scalability in the Myeloid dataset. Five low-represented cell types with fewer than 100 reference cells were considered: cDC2_CXCL9 (25 cells), cDC2_ISG15 (53), pDC_LILRA4 (59), cDC3_LAMP3 (94), and cDC1_CLEC9A (98) (Additional file 1: Table 5). We compared per-class recall between federated and centralized models across four federated scenarios (Additional file 3: Figures S13-S14). In Top5, FedAvg–SMPC (three epochs, 17 rounds) achieved the same overall recall as the centralized model (0.44), with gains of 2–18% recall for cDC1_CLEC9A, cDC3_LAMP3, and cDC2_CXCL9, but a 17% decrease for cDC2_ISG15. In Top10, FedProx–SMPC (*μ = 0.01,* one epoch, two rounds) yielded slightly lower overall recall (0.42 vs. 0.44); recall for pDC_LILRA4 and cDC1_CLEC9A was unchanged, increased by 3% for cDC3_LAMP3, and remained at 0 for cDC2_ISG15. In Top20, FedProx–SMPC (*μ = 0.01*, one epoch, 38 rounds) slightly improved overall recall (0.45 vs. 0.44), with stable performance for cDC1_CLEC9A, cDC2_ISG15, and pDC_LILRA4, and gains for cDC3_LAMP3 and cDC2_CXCL9. In Top30, FedProx–SMPC (*μ = 0.01*, one epoch, 180 rounds) further increased overall recall (0.46 vs. 0.44), with a 73% gain for cDC2_CXCL9, a 2% improvement for cDC3_LAMP3, a 2% decline for cDC1_CLEC9A, and unchanged recall for cDC2_ISG15. Across scalability scenarios, federated training maintained competitive overall and per-class recall for most low-represented cell types, with the exception of cDC2_ISG15 in Top5 and Top10 scenarios (Additional file 3: Fig. S14).

Privacy-preserving federated reference mapping matched the centralized baseline accuracy across all configurations, with the “rest” client outperforming centralized and federated models in Top5–Top20 but underperforming in Top30 (the most realistic federated scenario) (Fig. 5c). Overall, the federated model matched or exceeded the centralized model across all metrics for all splits, except for accuracy, recall, and macro-F1 in Top30, where the gap was limited to 0.01 (Additional file 3: Figures S11, S15).

### Privacy-preserving federated analysis exhibits heightened sensitivity to batch effects, yet attains performance comparable to the centralized baseline following batch effect correction

**Fig. 6:**
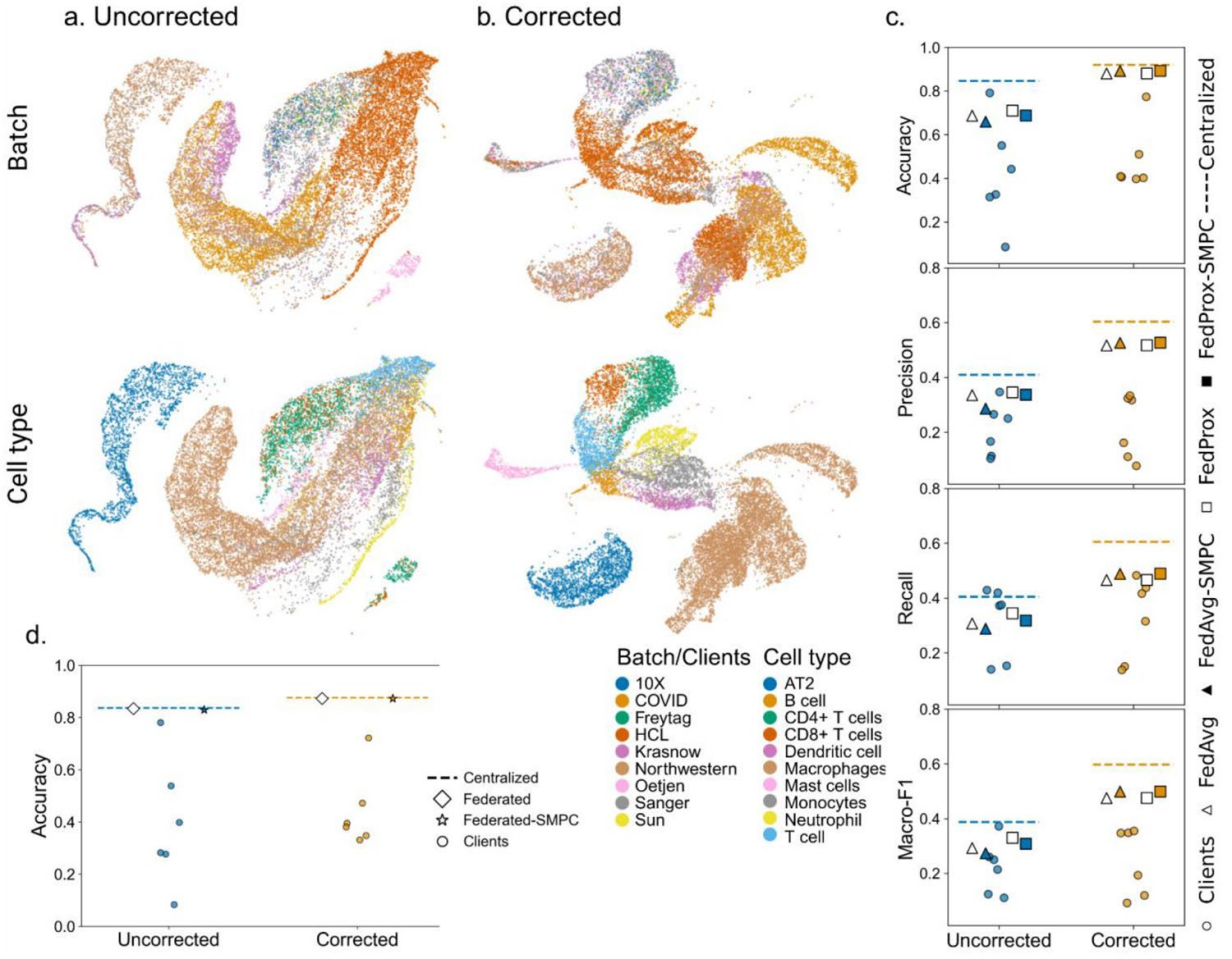
Batch effect correction of the Covid-19 dataset improves centralized and federated analysis and reduces performance gaps across training settings under a fixed training budget. (**a**) UMAP visualizations of a subset of the Covid-19 dataset colored by batch (top) and cell type (bottom) prior to correction. (**b**) UMAP after batch effect correction, demonstrating improved integration across batches and clearer cell type clustering. (**c**) Cell type classification performance comparing centralized, client-level, and federated models (FedAvg, FedAvg-SMPC, FedProx, FedProx-SMPC) trained for one epoch over 20 rounds on uncorrected vs. corrected data, evaluated by accuracy, precision, recall, and macro-F1. (**d**) Accuracy gain of centralized and federated (with and without SMPC) reference mapping after batch effect correction compared to client-level performance

We constructed a six-client federated scenario on the Covid-19 [44] dataset, which contains nine batches in total. Each client received one batch (10X, Covid, HCL, Northwestern, Oetjen, Sanger) with three batches held out as query data (Freytag, Krasnow, and Sun) (Additional file 1: Table 9). To illustrate the batch effect, we generated UMAP visualizations of the top ten most abundant cell types in the Covid-19 dataset (Fig. 6a). By reducing the number of displayed cell types and using distinguishable colors, we observed that in the uncorrected embeddings, cells cluster by batch rather than by underlying biology. For example, the HCL, Krasnow, and Northwestern batches are separated within the Macrophage population; similarly, Northwestern and Krasnow separate within the AT2 population, and Oetjen separates from the mixed group of 10X, Freytag, Krasnow, and Sun batches within CD4+ T cells (Fig. 6a). A complete UMAP visualization including all cell types shows a similar batch effect for smaller populations (Additional file 3: Fig. S16). We applied scGen with default parameters (see Methods) to correct for batch effects, considering all nine batches in the dataset. This correction resulted in improved mixing of the HCL, Krasnow, and Northwestern batches, as well as better separation between Macrophages and Monocytes (Fig. 6b). Overall, while batch mixing was only partially improved, the distinguishability of cell types was enhanced across all abundant populations, including Dendritic cells, Monocytes, and Neutrophils.

To assess the impact of batch effects on downstream analysis using the scGPT foundation model, we compared the performance of centralized, client-level, and federated models on both uncorrected and batch-corrected data using a fixed training budget of one local epoch and 20 communication rounds. On uncorrected data, federated models lagged substantially behind centralized performance (Fig. 6c). For example, FedProx-SMPC achieved an accuracy of 0.69 compared to 0.85 for centralized training, while some individual clients outperformed both federated and centralized models for specific metrics (Fig. 6c). Following batch effect correction, centralized performance improved across all metrics, with gains of 7–21% in accuracy, precision, recall, and macro-F1. Notably, the best privacy-preserving federated model (FedAvg-SMPC trained for one epoch over 61 rounds) closely matched centralized performance by reaching 0.93 accuracy, while extended training of up to 83 rounds reduced gaps in other metrics to less than 4% (Supplementary Figure S9). Furthermore, batch effect correction improves the reference mapping performance for both centralized and federated analyses across various metrics, while the privacy-preserving federated method consistently matches the centralized performance on both uncorrected and corrected data (Fig. 6d; Additional file 3: Fig. S17).

Throughout this study, we used federated weighted-average binning without SMPC protection. To assess whether the choice of binning strategy affected the reported results, we implemented and evaluated five binning strategies within the Clifti-GPT workflow: (i) local non-federated scGPT [5] quantile binning, performed independently within each client; (ii) federated weighted-average binning, in which local client bin edges are averaged with weights proportional to the number of cells; (iii) SMPC-protected federated weighted-average binning; (iv) federated histogram binning, which estimates global bin edges from the pooled empirical distribution using aggregated client histograms; and (v) SMPC-protected federated histogram binning. These strategies differed in how strongly bin assignments were associated with client identity and in the extent to which discretization amplified cross-client distributional heterogeneity. Nevertheless, the Clifti-GPT annotation accuracies were broadly similar across binning strategies for HP, LUNG, CL, MS, and Myeloid-top5 (Additional file 3: Fig. S18), reproducing the main best-accuracy trends reported in Fig. 2b.

To evaluate the practical deployment viability of privacy-preserving Clifti-GPT, we systematically profiled cryptographic wall-clock overhead across individual federated workflows using a controlled, GPU-accelerated CrypTen SMPC framework with three parties (Additional file 3: Fig. S19). Both secure model fine-tuning aggregation and federated binning were lightweight in absolute runtime. For a model with $\left| \theta \right| = {10^6}$ trainable parameters, secure aggregation completed within $\sim {31}{-}{\,36ms}$ across $C \in \left\{ {2,3,5} \right\}$ clients with $\sim 43--112 \times$ overhead relative to plaintext execution but remaining tolerable in absolute time (Additional file 3: Table 1). Similarly, downstream federated binning operations proved highly efficient; across $C \in \left\{ {2,5,10} \right\}$ clients, secure weighted-average binning completed in $\sim {4}{-}{19\,ms}$, while histogram-based binning executed in $\sim {9}{-}{19\,ms}$ (Additional file 3: Table 3). In contrast, secure KNN for reference mapping emerged as the dominant system bottleneck, scaling sharply with the number of query cells ${n_q}$ and reference cells ${n_r}$ (Additional file 3: Fig. S19b). Under SMPC, computing encrypted Euclidean-distances computations and secure comparison-based reductions introduced substantial latency, with relative runtime overheads of $\sim 11k{-}70k$ compared to plaintext execution. For a fixed ${n_q} = 2000$ query cells and *k = 10* neighbors, ten-fold increase of the reference set from ${n_r} = {10^3}$ to ${n_r} = {10^4}$ increased the median secure wall-clock time from $\sim 1.4\,min$ to $\sim 7.5\,min$ at *C = 5* clients (Additional file 3: Table 2). Consequently, the primary infrastructure and runtime bottleneck for privacy-preserving single-cell federated workflows is secure cell-to-atlas distance matching (KNN), rather than model-weight sharing or statistical binning (Additional file 3: Fig. S19c).

## Discussion

We present the first, to our knowledge, privacy-preserving federated fine-tuning and inference framework for foundation models (FMs) in scRNA-seq, providing a robust and scalable solution for distributed transcriptomic analysis. While FMs, as largely pre-trained models, offer strong representational capabilities, task-specific fine-tuning, often through the addition of specialized layers such as a classification head for cell type annotation, remains crucial for optimal performance in downstream applications. Notably, FMs can also be applied in a zero-shot setting without additional training, leveraging their rich embeddings to mitigate representation issues that hinder inference. In such cases, FM embeddings can be used directly for tasks like reference mapping or perturbation prediction by identifying the most similar reference profiles to a query sample. However, when scRNA-seq data are distributed across institutions, privacy-preserving federated learning enables zero-shot inference without centralizing sensitive genomic data. Given the stringent requirements of regulations such as GDPR [18] and institutional governance frameworks, centralizing such data, despite its typical accuracy advantage, is often infeasible. Meanwhile, traditional client-level training on local datasets may yield suboptimal performance due to dataset size limitations and sampling biases (Fig. 1d).

Against this backdrop, our proposed framework, Clifti-GPT, bridges the gap between centralized performance and decentralized constraints by securely aggregating model updates from distributed datasets using additive secret sharing. We evaluate multiple aggregation strategies, including FedAvg and FedProx, and examine the impact of SMPC-enabled aggregation on performance. Importantly, these experiments should be interpreted as a bounded sensitivity analysis rather than as a protocol for secure hyperparameter optimization. We used fixed reference budgets, including one local client epoch over 20 communication rounds, to compare federated configurations across datasets while avoiding extensive dataset-specific tuning. In centralized training, one epoch denotes a full pass over the pooled training data, whereas in federated training one communication round consists of local optimization at participating clients followed by aggregation of their updates. Thus, the number of communication rounds is not directly comparable to the number of centralized epochs. When Clifti-GPT reaches a specified fraction of centralized performance in a small number of communication rounds, this indicates communication-round efficiency relative to the evaluated centralized reference, not equivalence to the same number of centralized training epochs. Our findings highlight that best-performing configurations within the evaluated grid depend on both the target metric (accuracy, precision, recall, macro-F1) and the dataset. In some cases, such as the Lung-Kim and CL datasets, the same hyperparameters yield competitive performance with centralized models across all metrics (Fig. 1e). However, for other datasets (e.g., HP and MS), best-performing configurations vary substantially across metrics (Fig. 1e; Additional file 3: Figures S3, S9), indicating that no single federated setting uniformly dominated across tasks. Through a systematic, metric-driven hyperparameter analysis, we found that increasing local computation at clients does not necessarily improve convergence (Fig. 2c), whereas fewer local epochs can be beneficial in highly heterogeneous scenarios, such as Myeloid-Top30 (Fig. 5b). We provide a consolidated summary of best-performing hyperparameter settings across datasets in Additional file 3: Fig. S7.

Beyond absolute performance, communication efficiency is a key determinant of practical federated learning deployments. The Clifti-GPT workflow demonstrates strong communication efficiency across diverse datasets and levels of data heterogeneity (Fig. 2d). For the MS, HP, and Myeloid (Top5) datasets, the federated model achieves at least 95% of the centralized model performance within fewer than eight communication rounds across multiple evaluation metrics. Moreover, 99% of the centralized baseline is reached within 12 rounds for all dataset–metric pairs, except for HP–accuracy and MS–recall (Additional file 3: Fig. S8), highlighting the framework’s ability to balance high performance with low communication cost, a critical requirement for real-world federated learning settings. Accordingly, Clifti-GPT is well suited for scenarios where minimizing communication overhead is critical without sacrificing performance. When hyperparameters are tuned via metric-driven optimization, the privacy-preserving Clifti-GPT framework consistently matches centralized model performance across all metrics for the CL, Lung-Kim, Myeloid-Top5, and Myeloid-Top20 datasets. For the remaining datasets, Clifti-GPT with SMPC achieves centralized-level performance for most metrics, with only minor deviations: up to a 2% accuracy drop on HP and Myeloid-Top30, a 1% precision drop for Covid-19, a 4% recall drop for Covid-19, MS, and Myeloid-Top10, and a 3% macro-F1 drop for Covid-19 and Myeloid-Top10. Overall, Clifti-GPT with SMPC remains within 2% accuracy and within 4% for all other metrics relative to the corresponding centralized baselines across all evaluated datasets and federated scenarios (Additional file 3: Fig. S9), underscoring its robustness across metrics, datasets, and federation scales.

To rigorously evaluate performance under realistic non-IID conditions, we designed pathologically heterogeneous federated scenarios using genuine biological, cohort, disease-related, patient/sample, study, and batch structure rather than random or artificially induced partitions. These controlled simulations were intended to approximate distributional heterogeneity encountered across institutions or studies while preserving a shared evaluation protocol for centralized, local, and federated baselines. To isolate the effects of federated training and SMPC-protected aggregation, we used centralized feature harmonization, gene alignment, and preprocessing to establish a common feature space. This design produces high-skew inter-client distributions in which low-represented cell-type preservation during model aggregation is particularly challenging: clients with narrow or skewed distributions can disproportionately influence model updates, slow convergence, or bias predictions toward overrepresented cell types. We emphasize, however, that this benchmarking design does not fully capture all operational features of a real multi-institutional deployment, where sites may differ in sample collection, sequencing protocols, gene annotation, quality-control thresholds, normalization workflows, metadata standards, compute infrastructure, governance policies, and consent constraints. Strict federated deployment will therefore require additional methods for privacy-preserving gene-space alignment, feature harmonization, HVG selection, quality control, normalization, and batch correction.

Accordingly, we distributed data on a per-batch or annotation-defined basis, assigning each client all cells from one experimental batch where applicable, except for the residual client in Myeloid Top5–Top20. As a result, cell type composition varied substantially across clients: some had a broad mix of common and low-represented types, whereas others were dominated by one or a few cell types. For instance, in the Covid-19 scenario, certain clients were dominated by abundant types, while others, such as Sanger, were enriched for low-abundance populations. Rare types in the reference were absent in multiple clients, further amplifying inter-client heterogeneity (Additional file 1: Table 9). Moreover, in Myeloid-Top5, the “rest” client was dominated by highly abundant types yet still contained all low-represented types from the reference (Additional file 1: Table 5). In Myeloid-Top10, abundant types were more evenly distributed, reducing extreme dominance, but low-represented cells in the “rest” client became minimal (Additional file 1: Table 6). In Top20 and Top30, both the distribution of abundant types and the sparsity of low-represented types among the clients became more pronounced (Additional file 1: Tables 7–8).

Under these heterogeneous conditions, we evaluated cell-type-wise classification performance as a descriptive, engineering-focused assessment of framework behavior rather than as a statistically powered superiority analysis or an exploration of downstream biological mechanisms. The primary objective was to characterize how foundation-model fine-tuning behaves across individual cell types when executed within an SMPC-protected, decentralized environment. Low-represented cell types were defined as classes with fewer than 100 total cells in the baseline reference data. In the MS dataset, the federated true positive rate (TPR) for Phagocytes (47 cells) was 0.84, corresponding to a change of −0.08 relative to the centralized benchmark. Conversely, in the HP dataset, the low-represented Mast cell population (25 cells) reached a federated TPR of 0.71 (Fig. 3; Additional file 1: Tables 1–2). These examples could indicate the class-specific performance trade-offs that can occur when secure optimization distributes predictive errors across structurally imbalanced client matrices, highlighting that local gains or drops reflect dataset-specific, metric-driven adaptations rather than generalizable superiority.

Scalability is a critical requirement for applying federated foundation models to scRNA-seq analysis in real-world settings, where data are often generated across numerous institutions, studies, or sequencing batches with highly variable sizes and cell-type compositions. In four scalability scenarios, we partitioned 30 reference batches from the Myeloid dataset into increasingly fine-grained client distributions (Top5, Top10, Top20, and Top30). In the most heterogeneous and realistic Top30 case, although the local model of client 12 achieved comparable accuracy due to a cell-type distribution similar to the query (Additional file 1: Table 8), it failed to match the centralized model on other metrics (Additional file 3: Fig. S12). Overall, local client-level models failed to match the centralized baseline, whereas Clifti-GPT with SMPC consistently maintained performance within a worst-case margin of −0.03 across all metrics (Additional file 3: Fig. S9). These results highlight Clifti-GPT’s ability to scale effectively to complex, multi-institutional scRNA-seq environments, delivering robust performance despite the diversity and fragmentation inherent to real-world data.

Foundation models, despite their rich pretrained embeddings, are still prone to batch effects, and this limitation can be more impactful in federated analysis due to the isolation of data across clients. In our experiment on the Covid-19 dataset, we observed batch effects across multiple batches and cell types, for example HCL, Krasnow, and Northwestern within Macrophages (Fig. 6a; Additional file 3: Fig. S16). To mitigate this, we applied scGen with default parameters, correcting batches in the direction of the batch containing the majority of cells for each cell type. Accordingly, we selected three batches as queries that did not contain the maximum number of cells for any cell type, to avoid data leakage from query to reference. As a result, this correction improved mixing within HCL, Krasnow, and Northwestern and enhanced the separation of almost all cell types (Fig. 6b; Additional file 3: Fig. S16). Furthermore, batch effect correction improved centralized cell type classification performance by 7–21% across various metrics compared to uncorrected data (Fig. 6c). Notably, batch effects had a stronger impact on the federated analysis, where the best federated model performed 16% less accurately than the centralized baseline on uncorrected data, emphasizing the susceptibility of federated models to this issue (Fig. 6c). After correction, the privacy-preserving federated model closed the gap, achieving 0.93 accuracy (Additional file 3: Fig. S9). Likewise, reference mapping performance also improved for both centralized and federated models (Fig. 6d; Additional file 3: Fig. S17), although the margin of improvement was more limited compared to fine-tuning (Additional file 3: Fig. S17). Overall, these findings demonstrate that batch effects had a greater impact on fine-tuning than on zero-shot applications, though correction enhanced performance in both settings. Our evaluation primarily addresses label and client-distribution heterogeneity in SMPC-protected federated workflows, whereas feature-level heterogeneity, including batch effects, remains an important direction for future privacy-preserving benchmarking. The batch-corrected COVID-19 experiment should therefore be interpreted as a sensitivity analysis of technical feature heterogeneity, not as part of the strict privacy-preserving federated workflow or evidence that Clifti-GPT solves federated batch correction. Batch correction was applied using default scGen settings without dataset-specific optimization, as the study focused on benchmarking Clifti-GPT rather than comparing centralized or federated [55] methods.

Beyond traditional fine-tuning, zero-shot applications of foundation models present unique opportunities and privacy risks in decentralized clinical environments. While centralized analyses can directly exploit rich foundation-model embeddings for downstream reference mapping, a federated setting requires raw data, embeddings, and intermediate statistics to remain protected to support institutional governance constraints. To address this setting, we introduce transferable inference as an end-to-end secure zero-shot workflow that goes beyond a simple federated KNN step. As detailed in Additional file 2, the pipeline keeps the embedded query, client-level KNN outputs, global neighbor-selection steps, and vote aggregation in secret-shared form across multiple steps without exposing plaintext intermediate values, except for the total reference-set size needed for global indexing. Specifically, ClientSetup (Algorithm 4) secret-shares each client’s reference embeddings, offset indices, and mapped labels; SecureKNN (Algorithm 5) performs distance computation, masked minimum selection, index extraction, and suppression on secret-shared values; and SecureVote (Algorithm 6) constructs the final votes directly from secret-shared neighbor indices and labels, revealing only the final prediction-relevant output. We use the term transferable inference to distinguish this cryptographically protected workflow from conventional federated KNN on raw data or plaintext embeddings, both of which can expose sensitive information during computation. By keeping embedding handling and downstream inference within the computational parties, Clifti-GPT transfers inference-relevant biological information across clinical cohorts while avoiding disclosure of raw data, plaintext embeddings, intermediate distances, neighbor identities, and localized voting statistics under the stated semi-honest threat model.

The zero-shot application of FMs in scRNA-seq analysis represents a major advance, enabled by improvements in model architectures and increasingly expressive embedding representations. These embeddings capture rich transcriptomic features that can be directly leveraged for a range of downstream analyses without task-specific fine-tuning, including reference mapping for cell type annotation, perturbation prediction, and reverse perturbation inference based on distance-driven comparisons between query and reference cells [5]. As embedding quality continues to improve, the potential impact of such zero-shot approaches is substantial, enabling faster, more generalizable, and resource-efficient workflows that can adapt to new datasets and analytical tasks. Consistent with this, comparing the centralized performance of fine-tuning vs. reference mapping for inferring cell types in the CL, Covid-Corrected, Lung-Kim, and Myeloid (all scenarios) datasets shows that although reference mapping trails fine-tuning across all metrics, the worst-case margin is only 0.07, underscoring the potential of zero-shot foundation models as a viable alternative to fine-tuning (Additional file 3: Figures S9, S11). A similar margin of difference between zero-shot and fine-tuning is observed across federated scenarios (Additional file 3: Figures S9, S11). Realizing the full potential of zero-shot FM applications in real-world settings requires addressing several challenges. For instance, while scalability and heterogeneity can affect performance, Clifti-GPT maintained consistent accuracy in Myeloid scenarios, with at most a 1% drop from the centralized baseline across all metrics (Additional file 3: Fig. S11). Furthermore, while batch effects remain a major source of variation that can confound embeddings, these can be mitigated through correction, as shown by our federated model matching centralized accuracy and staying within a 5% margin on precision, recall, and macro-F1 for the Covid-19 dataset (Additional file 3: Fig. S11).

From a privacy perspective, Clifti-GPT uses additive secret sharing to limit direct plaintext exposure of client-level quantities during both federated fine-tuning and zero-shot reference mapping. In fine-tuning, client updates are split into shares before secure aggregation; in transferable inference, protected quantities such as embeddings, distance computations, neighbor-selection statistics, and voting information are handled in secret-shared form until the intended reveal step. This design is particularly relevant for foundation-model workflows, where high-dimensional updates or embeddings may encode rare or donor-specific signals and could otherwise contribute to reconstruction, model-inversion, or membership-inference risks. Additive secret sharing does not intentionally add privacy noise; rather, it preserves the intended computation up to implementation-level numerical effects while reducing direct exposure of protected intermediate quantities during the secure protocol.

These protections should be interpreted under a semi-honest, honest-but-curious SMPC threat model, in which participating clients, the coordinator, and computational parties are assumed to follow the prescribed protocol but may inspect the information available to them during execution [31,34]. Under this model, SMPC protects the computation process by secret-sharing protected client-level quantities, including plaintext model updates, embeddings, neighbor-selection statistics, and voting information, so that no single party observes another site’s protected inputs or intermediate values in plaintext during the secure protocol. This protection is distinct from ordinary data locality in federated learning: keeping raw data on site does not by itself prevent leakage from intermediate updates, embeddings, aggregate outputs, or final models, whereas SMPC reduces exposure of protected intermediate quantities during computation [32, 41]. At the same time, the stated threat model does not eliminate all privacy or security risks. The aggregate update, final global model, predictions, reports, or other released outputs remain legitimate outputs of the workflow and may still raise output-privacy concerns, including model inversion, membership inference, reconstruction, or other leakage risks [29, 30, 56]. These output-level risks are conceptually separate from the SMPC guarantee that intermediate client-level quantities are protected during computation. Similarly, the current framework does not claim robustness to malicious clients, collusion, poisoning, Byzantine behavior, active protocol deviations, or unintentional data corruption; such settings require complementary defenses such as malicious-secure SMPC, Byzantine-robust aggregation, poisoning defenses, anomaly detection, auditing, and deployment-specific governance controls [57–59]. Therefore, Clifti-GPT should be viewed as a protected computation layer for federated foundation-model fine-tuning and transferable inference under the semi-honest SMPC model, rather than as a complete solution to output privacy, adversarial robustness, or institutional deployment governance. We did not empirically evaluate attack-specific adversarial scenarios, such as model inversion, membership inference, reconstruction, poisoning, or malicious-client behavior [29, 30, 56, 59]. Such analyses are complementary to the present work, whose privacy claim is limited to SMPC-protected computation-process privacy under the stated semi-honest threat model [31, 34].

Clifti-GPT is designed to preserve privacy during reference mapping under the stated semi-honest SMPC threat model by maintaining intermediate quantities in secret-shared form until the intended reveal step. Clients’ shared data are used for reference mapping without disclosing cell-level information in plaintext, including raw expression profiles, plaintext embeddings, nearest-neighbor identities, or voting information, to any single party during the secure computation. By avoiding disclosure of intermediate neighbors and voting quantities, this design limits leakage about client-specific reference-cell contributions and cell-type availability during the secure protocol, which is important for governance-sensitive settings involving rare or novel cell types. Under the adopted semi-honest SMPC assumptions, the identities of the nearest reference cells to query cells, their associated labels, and client-level voting contributions remain secret-shared until the intended reveal step and are not revealed to any single party during secure computation. Similarly, query samples are kept in secret-shared form, so reference holders and other parties do not observe their contents or mapping relationships during secure processing. Together, these design choices enable collaborative reference mapping, nearest-neighbor search, and voting to be performed without exposing intermediate quantities outside the secure computation protocol, preserving the confidentiality of both reference and query intermediate quantities during secure computation.

We also analysed five binning strategies within the Clifti-GPT workflow and found broadly similar annotation accuracies across the five scGPT annotation datasets (Additional file 3: Fig. S18). This finding suggests that the principal downstream annotation performance is not driven by a single binning implementation. At the same time, the histogram-based strategies may provide a better alternative to weighted averaging of local quantiles under non-IID client distributions, and the SMPC-protected variants provide a privacy-preserving approach to federated binning.

The integration of SMPC introduces workflow-specific cryptographic overhead that must be considered separately from communication efficiency. Our simulated CrypTen profiling shows that secure aggregation for federated binning and fine-tuning has moderate overhead, whereas secure KNN for reference mapping stands as the dominant cryptographic bottleneck due to requiring repeated encrypted distance computation, secure comparison, masked minimum selection, index extraction, and minimum suppression over query and reference embeddings (Additional file 3: Fig. S19 and Tables 1–3). This extreme performance divergence stems from the underlying algorithmic primitives: model-weight sharing and weighted-average statistical binning rely on local, non-interactive operations on secret shares, whereas secure KNN demands an exhaustive sequence of highly interactive, non-linear operations. These include encrypted multidimensional distance computations, iterative secure comparisons, masked minimum selections, index extractions, and minimum suppressions over query and reference embeddings, each requiring expensive network communication rounds between the computing parties. These profiling results should be interpreted as a controlled characterization of cryptographic kernel overhead rather than as a complete deployment benchmark. Real multi-institutional deployment will depend on network latency, bandwidth, hardware, the number of SMPC parties, implementation choices, and whether cryptographic computation is hosted by clinical sites or delegated to dedicated computational parties [41]. Importantly, once client-side quantities are secret-shared, subsequent secure operations can be carried out by the designated SMPC computing parties without revealing plaintext intermediate values to any entity under the stated semi-honest model [34, 41]. This decoupled architecture can significantly alleviate the computational burden on data-contributing hospitals; clinical sites primarily execute local preprocessing, plaintext embedding generation, and initial secret sharing, while delegating the highly expensive secure aggregation and secure KNN kernels to dedicated, high-performance computational nodes. Nevertheless, practical deployment will require site-specific benchmarking and infrastructure planning.

This study underscores the potential of foundation models to transform single-cell transcriptomics by combining rich, pre-trained embeddings with adaptable fine-tuning strategies. As future FMs are trained on larger and more diverse biological corpora with improved embedding architectures, their ability to support accurate zero-shot inference and efficient domain-specific fine-tuning will only increase. At the same time, the sensitive and often regulated nature of single-cell data creates a growing demand for federated approaches that allow model improvement without centralizing patient-level data. Our framework demonstrates how such models can be integrated into distributed workflows using additive secret sharing, addressing both the technical challenges of scalability and data heterogeneity, and the practical requirements of privacy-preserving computation, data governance, and GDPR-relevant design principles. By enabling robust, metric-driven performance in settings with extreme non-IID distributions, this work provides a blueprint for adopting next-generation FMs in a privacy-preserving and communication-efficient manner. However, developing a comprehensive, end-to-end pipeline will require extending this framework to include additional preprocessing and downstream components, such as feature harmonization, HVG selection, and batch-effect correction. Each of these modules introduces unique cryptographic, statistical, and deployment challenges that could act as confounding factors when comparing baseline and secure models. Addressing these challenges, alongside implementing statistical testing and biological interpretation for rare cell types, remains a key direction for future work. Ultimately, this framework sets the stage for scalable, collaborative analysis pipelines that respect institutional boundaries while leveraging the full capabilities of emerging foundation models.

## Electronic supplementary material

Below is the link to the electronic supplementary material.

Supplementary Material 1

Supplementary Material 2

Supplementary Material 3

## Data Availability

No new datasets were generated during the current study. All datasets analyzed in this study were previously published and are cited in the article.The preprocessed datasets are available at the Zenodo repository specified in the Code availability section.

## Code availability

All datasets used in this study were published in previously cited papers. The preprocessed datasets are available at 10.5281/zenodo.20491148 and the initialized PyTorch models for reproducibility are available at 10.5281/zenodo.20489646. The Clifti-GPT source code is available at https://github.com/Mohammad-Bakhtiari/clifti-GPT, under the Apache 2.0 license.
