# Supplementary material for: Clifti-GPT: privacy-preserving federated fine-tuning and transferable inference of foundation models on clinical single-cell data
Source: BioData Min. 2026 Aug 5;19:58. doi: 10.1186/s13040-026-00582-w (PMC13445909; doi:10.1186/s13040-026-00582-w)
Supplement: Supplementary file 1 [file 13040_2026_582_MOESM2_ESM.pdf]

Supplementary: Method

1 Federated binning

Algorithm 1 Federated weighted-averag binning

Definitions:
$\mathcal{P} \leftarrow$ number of SMPC parties
$\langle \cdot \rangle \leftarrow [\![\cdot]\!]_{\mathcal{P}}$ (secret-sharing)
$(\!| \cdot |\!)_{\mathcal{P}}$ (SMPC reveal)
Input: Local data D_i across C clients, number of bins B
for each client $i \in \{1, 2, \ldots, C\}$ **do**
    $B_i \leftarrow \text{Quantile}(D_i^{>0}, \text{linspace}(0, 1, B-1)), \quad n_i \leftarrow |D_i^{>0}|$ ▷ $B_i \in \mathbb{R}^{B-1}$
end for
if secret share **then**
    for each client i: $\langle B_i \rangle \leftarrow [\![B_i]\!]_{\mathcal{P}}, \quad \langle n_i \rangle \leftarrow [\![n_i]\!]_{\mathcal{P}}$ ▷ Secret-share
    $\langle B\_global \rangle \leftarrow \sum_i \langle n_i \rangle \langle B_i \rangle \ / \ \sum_i \langle n_i \rangle$
    $B\_global \leftarrow (\!| \langle B\_global \rangle |\!)_{\mathcal{P}}$
else
    $B\_global \leftarrow \frac{\sum_{i=1}^{C} n_i B_i}{\sum_{i=1}^{C} n_i}$ ▷ Share plaintext
end if
$B\_global \leftarrow \text{Quantile}(B\_global, \text{linspace}(0, 1, B-1))$
Each client bins its local data using B_global

Algorithm 2 Federated histogram binning

Definitions:
  $\mathcal{P} \leftarrow$ number of SMPC parties
  $\langle\cdot\rangle \leftarrow [\![\cdot]\!]_{\mathcal{P}}$ (secret-sharing)
  $(\!|\cdot|\!)_{\mathcal{P}}$ (SMPC reveal)
Input: local data $\{D_i\}_{i=1}^{C}$, bin count B, grid resolution M
Round 1: Max-expression aggregation
for each client $i \in \{1, \ldots, C\}$ **do**
  $m_i \leftarrow \max(D_i^{>0})$
end for
if secret share **then**
  for each client i: $\langle m_i \rangle \leftarrow [\![m_i]\!]_{\mathcal{P}}$
  $max\_expr \leftarrow (\!|\max_{i\in\{1,\ldots,C\}} \langle m_i \rangle|\!)_{\mathcal{P}}$ ▷ Reveal global max only
else
  $max\_expr \leftarrow \max_{i\in\{1,\ldots,C\}} m_i$ ▷ Share plaintext maximum expr
end if
$g \leftarrow \text{linspace}(0,\, max\_expr,\, M+1)$
Round 2: Global histogram aggregation
for each client $i \in \{1, \ldots, C\}$ **do**
  $H_i \leftarrow \text{histogram}(D_i^{>0},\, g) \in \mathbb{N}^M, \quad n_i \leftarrow |D_i^{>0}|$
end for
if secret share **then**
  for each client i: $\langle H_i \rangle \leftarrow [\![H_i]\!]_{\mathcal{P}}, \quad \langle n_i \rangle \leftarrow [\![n_i]\!]_{\mathcal{P}}$
  $\langle H \rangle \leftarrow \sum_{i=1}^{C} \langle H_i \rangle, \quad \langle N \rangle \leftarrow \sum_{i=1}^{C} \langle n_i \rangle$ ▷ Secret-share
else
  $H \leftarrow \sum_{i=1}^{C} H_i, \quad N \leftarrow \sum_{i=1}^{C} n_i$ ▷ Share plaintext H_i & n_i
end if
$p \leftarrow \text{linspace}(0,\, 1,\, B-1)$
if secret share **then**
  $\forall m \in \{1, \ldots, M\}: \ \langle S_m \rangle \leftarrow \sum_{m'=1}^{m} \langle H_{m'} \rangle$
  for $j = 1$ **to** $B-1$ **do**
    $\langle t_j \rangle \leftarrow \max(p_j \cdot \langle N \rangle,\, 1)$
    $\langle \mu_j \rangle \leftarrow \text{geMask}(\langle S \rangle,\, \langle t_j \rangle)$
    $\langle \tilde{\mu}_j \rangle \leftarrow [\,0,\ \langle \mu_j \rangle_{1:M-1}\,]$
    $\langle e_j \rangle \leftarrow \langle \mu_j \rangle \odot (1 - \langle \tilde{\mu}_j \rangle)$
    $\langle B\_global \rangle_j \leftarrow \langle e_j \rangle\, g_{2:M+1}$
  end for
  $B\_global \leftarrow (\!|\langle B\_global \rangle|\!)_{\mathcal{P}}$
else
  $\forall m \in \{1, \ldots, M\}: \ S_m \leftarrow \sum_{m'=1}^{m} H_{m'}$
  for $j = 1$ **to** $B-1$ **do**
    $t_j \leftarrow \max(p_j \cdot N,\, 1)$
    $m_j^{\star} \leftarrow \min_{m\in\{1,\ldots,M\}:\, S_m \geq t_j} m$
    $B\_global_j \leftarrow g_{m_j^{\star}+1}$
  end for
end if
Each client bins its local data using B_global

2 Federated reference mapping

Algorithm 3 Federated Zero-Shot Reference Mapping

Input: $\{\mathcal{R}_i\}_{i=1}^{C}$, $\mathcal{Q}$, $\mathcal{F}$, k
$\mathcal{Q}' \leftarrow \mathcal{F}(\mathcal{Q})$
for each client $i \in \{1, \ldots, C\}$ **do**
    $\mathcal{R}'_i \leftarrow \mathcal{F}(\mathcal{R}_i)$
    $(\mathcal{D}_i, \mathcal{I}_i) \leftarrow \|\mathcal{Q}' - \mathcal{R}'_i\|$ ▷ keep top-k per client
end for
$k\_nearest_j \leftarrow \mathrm{TopK}\big(\{\mathcal{I}_i[j, 1 \ldots k]\}_{i=1}^{C}\big) \quad (\forall j \in \mathcal{Q})$
for each client $i \in \{1, \ldots, C\}$ **do**
    $\mathcal{V}_i[j] \leftarrow \mathcal{L}_i\big[\mathcal{I}_i[j]\big] \quad (\forall j \in k\_nearest_j)$
end for
$Predictions \leftarrow \mathrm{mode}\{\mathcal{V}_i[j]\}_{i=1}^{C}$ for each $j \in \mathcal{Q}$

2.1 Secure federated reference mapping

Algorithm 4 Secure Federated Zero-Shot Reference Mapping

Definitions:
$\mathcal{P} \leftarrow$ number of SMPC parties
$\langle\cdot\rangle \leftarrow [\![\cdot]\!]_{\mathcal{P}}$ (secret-sharing)
$(\!|\cdot|\!)_{\mathcal{P}}$ (SMPC reveal)
$\Lambda \leftarrow$ large masking constant
$\Phi\colon \mathcal{L} \rightarrow \{1, \ldots, |\mathcal{L}|\}$ (label→index map)
shapes: $\langle\mathcal{D}\rangle \in \mathbb{F}^{|\mathcal{Q}|\times(Ck)}$, ...
Input: query Q, references $\{\mathcal{R}_i\}_{i=1}^{C}$, model $\mathcal{F}$, neighbor count k
$\langle\mathcal{Q}\rangle \leftarrow [\![\mathcal{F}(\mathcal{Q})]\!]_{\mathcal{P}}$ ▷ Secret-share embedded query
$\mathcal{O} \leftarrow (\!|\sum_{i=1}^{C}\langle n_i\rangle|\!)_{\mathcal{P}}$ ▷ Reveal total number of samples as offset
$\mathcal{L} = \bigcup_{i=1}^{C} l_i$ ▷ All unique labels
$\phi = \left[\Phi(\ell) : \ell \in \mathcal{L}\right]$ ▷ label index-vector
$\langle\mathcal{D}\rangle \leftarrow \mathbf{0}^{|\mathcal{Q}|\times(C\,k)}, \quad \langle\mathcal{I}\rangle \leftarrow \mathbf{0}^{|\mathcal{Q}|\times(C\,k)}$
for $i = 1$ **to** C **do** ▷ Local secure k-NN per client
    ClientSetup($\mathcal{F}$, Φ, $(i-1)\mathcal{O}$)
    $(\langle d\rangle, \langle ind\rangle) \leftarrow$ SecureKNN$(\langle\mathcal{Q}\rangle, k)$
    $\langle\mathcal{D}\rangle_{:,(i-1)*k:i*k} \leftarrow \langle d\rangle, \quad \langle\mathcal{I}\rangle_{:,(i-1)*k:i*k} \leftarrow \langle ind\rangle$
end for
$\langle\mathcal{N}\rangle \leftarrow \mathbf{0}^{|\mathcal{Q}|\times k}$
for $j = 1$ **to** k **do**
    $\langle\mathcal{M}\rangle = \text{minMask}(\langle\mathcal{D}\rangle)$
    $\langle\mathcal{N}\rangle_{:,j} = \langle\mathcal{M}\rangle\,\langle\mathcal{I}\rangle$
    $\langle\mathcal{D}\rangle \mathrel{+}= \Lambda\,\langle\mathcal{M}\rangle$
end for
for $i = 1$ **to** C **do**
    $\langle V_i\rangle = \text{SecureVote}(\langle\mathcal{N}\rangle, |\mathcal{Q}|, |\mathcal{L}|)$
end for
$\langle\mathcal{V}\rangle \leftarrow \sum_{i=1}^{C}\langle V_i\rangle$
$\langle M\rangle \leftarrow \text{maxMask}(\langle\mathcal{V}\rangle)$ ▷ secret-shared one-hot of highest-vote class
$M^{\text{plain}} \leftarrow (\!|\langle M\rangle|\!)_{\mathcal{P}}$
$\text{predictions} = \sum_{\ell=1}^{|\mathcal{L}|} M^{\text{plain}}_{:,\ell}\,\phi_\ell$
return predictions

Algorithm 5 ClientSetup

Precondition: $\mathcal{R}$, l, $\mathcal{P}$
Input: Model $\mathcal{F}$, Offset $\mathcal{O}$, label-to-index map Φ
$\langle\mathcal{R}\rangle \leftarrow [\![\mathcal{F}(\mathcal{R})]\!]_{\mathcal{P}}$ ▷ Secret-share reference embeddings
$\langle r\rangle \leftarrow [\![[0, 1, \ldots, |\mathcal{R}|-1] + \mathcal{O}]\!]_{\mathcal{P}}$ ▷ Secret-share index vector
$\ell \leftarrow [\Phi(l_r) : \forall\, r \in \{0, \ldots, |R|-1\}]$ ▷ map local labels
$\langle\ell\rangle \leftarrow [\![\ell]\!]_{\mathcal{P}}$ ▷ secret-share mapped label indices
$\Lambda \leftarrow$ large mask constant

Algorithm 6 SecureKNN

Precondition: $\langle \mathcal{R} \rangle$, $\langle r \rangle$, Λ
Input: secret-shared query $\langle \mathcal{Q} \rangle$, neighbor count k
$\langle \mathcal{D}^{(0)} \rangle = (\langle \mathcal{Q} \rangle \circ \langle \mathcal{Q} \rangle)\, \mathbf{1}_{|\mathcal{R}|}^{T} + \mathbf{1}_{|\mathcal{Q}|}\, (\langle \mathcal{R} \rangle \circ \langle \mathcal{R} \rangle)^{T} - 2\, \langle \mathcal{Q} \rangle \, \langle \mathcal{R} \rangle^{T}$
$\langle \mathcal{T} \rangle \leftarrow \mathbf{0}^{\,|\mathcal{Q}| \times k}, \quad \langle \mathcal{I} \rangle \leftarrow \mathbf{0}^{\,|\mathcal{Q}| \times k}$
for $j = 1$ **to** k **do**
    $\langle \mathcal{M} \rangle = \mathrm{minMask}(\langle \mathcal{D}^{(j-1)} \rangle)$ ▷ One-hot argmin
    $\langle \mathcal{T} \rangle_{:,j} = (\langle \mathcal{M} \rangle \odot \langle \mathcal{D}^{(0)} \rangle)\, \mathbf{1}_{|\mathcal{R}|}$ ▷ Hadamard-product
    $\langle \mathcal{I} \rangle_{:,j} = \langle \mathcal{M} \rangle \, \langle r \rangle$
    $\langle \mathcal{D}^{(j)} \rangle = \langle \mathcal{D}^{(j-1)} \rangle + \Lambda \, \langle \mathcal{M} \rangle$ ▷ Suppress the chosen minima
end for
return $(\langle \mathcal{T} \rangle,\ \langle \mathcal{I} \rangle)$

Algorithm 7 SecureVote

Precondition: $\langle r \rangle$, $\langle \ell \rangle$
Input: Global neighbors $\langle \mathcal{N} \rangle$, number of query samples n_q,number of unique labels n_l.
$\langle V \rangle \leftarrow \mathbf{0}^{\,n_q \times n_l}$
for $j = 1$ **to** k **do**
    $\langle M \rangle \leftarrow \mathrm{eqMask}(\, \langle \mathcal{N} \rangle_{:,j}, \langle r \rangle)$ ▷ secret-shared equality check
    $\langle v \rangle \leftarrow \langle M \rangle \, \langle \ell \rangle$
    $\langle V \rangle \mathrel{+}= \ \mathrm{oneHot}(\langle v \rangle,\ n_l)$ ▷ $\langle v \rangle = 0 \rightarrow$ *no vote*
end for
return $\langle V \rangle$

**Notation**	**Description**
k	Number of nearest neighbors.
C	Number of clients (parties) in the federation.
i	Client i index.
$\mathcal{F}$	Shared embedding model.
$[\![\cdot]\!]_{\mathcal{P}}$	Secret-sharing operator among $\mathcal{P}$ parties.
$\mathcal{P}$	Number of SMPC parties.
$\langle\cdot\rangle$	Secret-sharing operand under the SMPC protocol.
$\mathcal{Q}$	Query dataset.
$\mathcal{R}$	Reference dataset.
$\mathcal{Q}'$	Embedded query.
$\langle\mathcal{Q}\rangle$	Secret-shared embedded query.
$\mathcal{R}'$	Embedded reference dataset.
$\langle\mathcal{R}\rangle$	Secret-shared reference data.
l_i	Client-i local labels.
$\mathcal{L}$	Global set of all unique labels.
$\mathcal{O}$	Global index offset.
Φ	Bijection from label strings to integer indices.
ϕ	Label index-vector.
r_i	Client-i offset indices.
$\langle r_i\rangle$	Secret-shared offset indices of client-i.
ℓ_i	Client-i mapped local labels.
$\langle\ell_i\rangle$	Secret-shared mapped label indices of client-i.
Λ	Large constant value.
$\langle\mathcal{D}^{(0)}\rangle$	Initial local distance matrix before masking.
$\langle\mathcal{D}^{(j)}\rangle$	local distance matrix after masking out the first j minima.
$\langle\mathcal{T}\rangle_{:,j}$	Distance vector for j-st nearest neighbor per query.
$\langle\mathcal{I}\rangle_{:,j}$	Index vector for j-st nearest neighbor per query.
$\langle\mathcal{D}\rangle$	Secret-shared query-reference distance matrix.
$\langle\mathcal{I}\rangle$	Secret-shared indices of reference samples in $\langle\mathcal{D}\rangle$.
$\langle\mathcal{N}\rangle$	One-hot secret indices of the global nearest neighbor.
$\langle v\rangle$	Client's secret-shared vote vector (per neighbor).
$\langle V_i\rangle$	Client-i's secret-shared vote matrix.
$\langle\mathcal{V}\rangle$	Aggregated secret vote matrix across all clients.
M^{plain}	One-hot matrix of indices of the most frequent votes.

Table 1: Notation table.

3 Simulated cryptographic overhead

We benchmarked the computational cost of three federated Clifti-GPT workflows: fine-tuning weight aggregation, federated reference mapping, and federated binning. For each workflow, we compared plaintext execution with CrypTen-based secure multi-party computation (SMPC)[1]. The goal of this analysis was to characterize measured cryptographic runtime overhead under controlled conditions, rather than to estimate analytical communication volume or real multi-site network performance. Accordingly, we report measured wall-clock timings and plaintext-versus-SMPC timing overheads. For each configuration, we recorded the median wall-clock time over five timed repetitions. All experiments were executed on a server with four NVIDIA Tesla T4 GPUs, each with approximately 16 GB of VRAM. SMPC benchmarks were executed on GPU with $\mathcal{P} = 3$ CrypTen computational parties, assigning one GPU to each party. This setup measures in-process cryptographic overhead and excludes real inter-site latency, bandwidth contention, and deployment-specific hardware heterogeneity. Timing overhead was defined as $t_{\text{SMPC}}/t_{\text{plain}}$.

Clifti-GPT fine-tuning. We ran 18 timed configurations corresponding to nine federated grid points: $C \in \{2, 3, 5\}$ clients crossed with three trainable-parameter counts, $|\theta| \in \{10^6, 10^7, 51{,}337{,}228\}$. Each configuration was evaluated under plaintext execution and CrypTen SMPC ($\mathcal{P} = 3$). The value $|\theta| = 51{,}337{,}228$ is the trainable parameter count obtained from the checkpoint used to instantiate the scGPT pretrained model on the Human Pancreas (HP) dataset; the 10^6 and 10^7 settings are synthetic state dictionaries of the same structural class, included to probe runtime scaling over larger model-update tensors. For each grid point, we timed one FedAvg aggregation step. Each timing is the median of five repetitions.

Clifti-GPT transferable inference (KNN). We ran 48 timed configurations corresponding to 24 federated grid points: $n_q \in \{500, 2000\}$ query cells, federation-wide reference sizes $n_r \in \{1{,}000, 5{,}000, 10{,}000\}$, client counts $C \in \{2, 5\}$, and neighborhood sizes $k \in \{5, 10\}$. Each configuration was evaluated under plaintext execution and SMPC. The embedding dimension was fixed at $d = 128$. Reference cells were partitioned evenly across clients ($n_{r,\text{local}} = \lfloor n_r/C \rfloor$). For each grid point, we timed one federated KNN pass: per-client distance computation and top-k selection, followed by a global top-k merge over all clients. The plaintext baseline uses the same GPU distance and top-k structure as the SMPC implementation, so the overhead ratios isolate the privacy-preserving cost on comparable hardware.

Clifti-GPT binning. We ran 12 timed configurations across federated simulations with $C \in \{2, 5, 10\}$ clients. For each value of C, we evaluated four aggregation strategies: weighted quantile averaging (plaintext and SMPC) and histogram-based binning (plaintext and SMPC). Bin geometry was fixed at 51 bins with histogram grid resolution $M = 4096$; each client contributed 100,000 synthetic non-zero expression values. For every configuration, we timed one federated bin-edge aggregation, either by merging locally computed quantile edges or by pooling secret-shared histograms and reading quantile cuts from the

aggregated CDF. Timing overhead for the SMPC variants is reported relative to the matching plaintext strategy at the same value of C.

References

[1] Knott, B. *et al.* Crypten: Secure multi-party computation meets machine learning. In *Advances in Neural Information Processing Systems* (2021).
