# Supplementary material for: Clifti-GPT: privacy-preserving federated fine-tuning and transferable inference of foundation models on clinical single-cell data
Source: BioData Min. 2026 Aug 5;19:58. doi: 10.1186/s13040-026-00582-w (PMC13445909; doi:10.1186/s13040-026-00582-w)
Supplement: Supplementary file 2 [file 13040_2026_582_MOESM3_ESM.pdf]

Supplementary: Figures

1 Clients data distribution

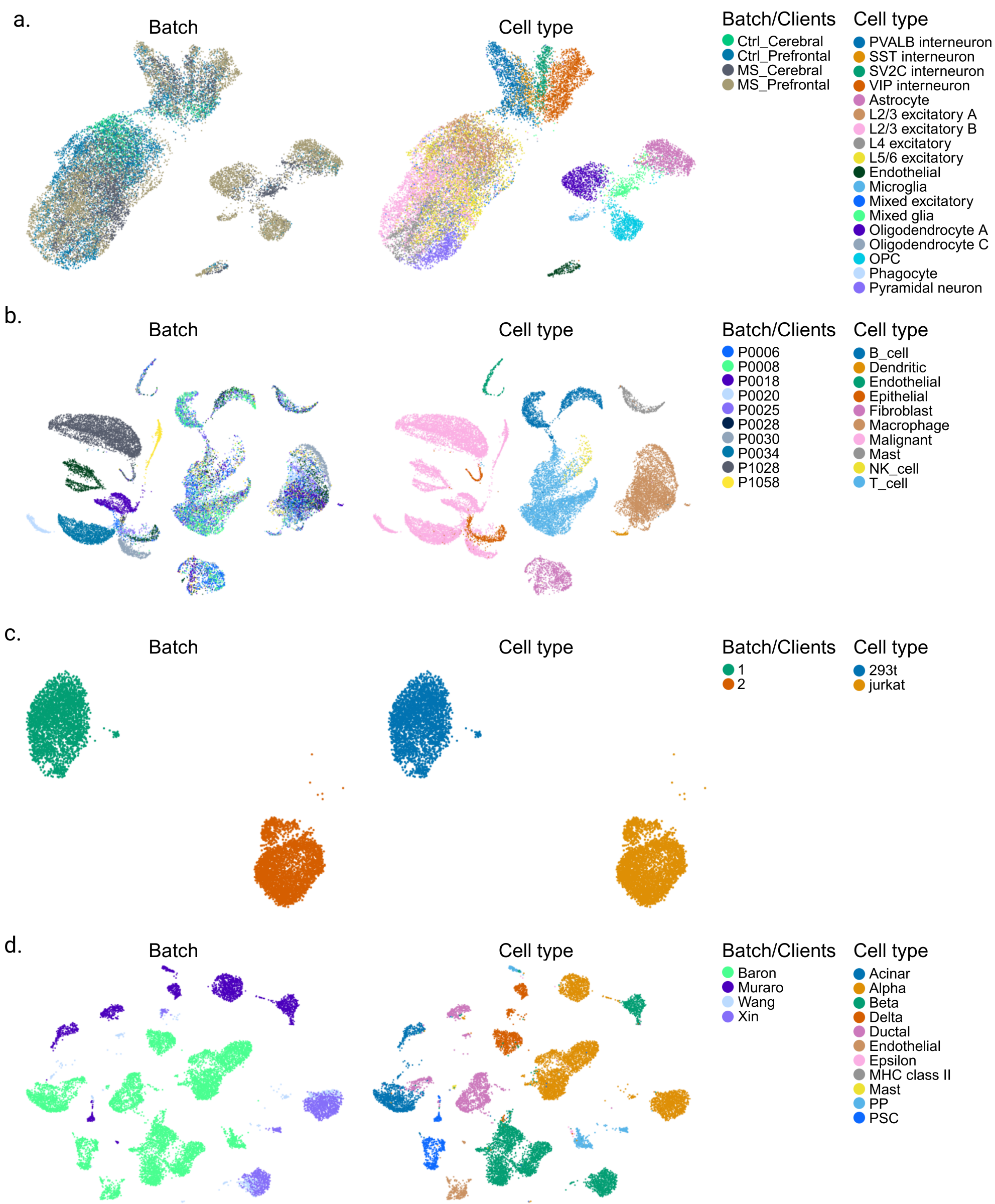

Fig S1: UMAP visualization showing batch and cell type distributions for the reference datasets. In the federated scenarios, each batch is assigned to a specific client: **a.** Multiple Sclerosis (MS) dataset. **b.** Lung-Kim dataset. **c.** CellLine (CL) dataset. **d.** Human Pancreas (HP) dataset.

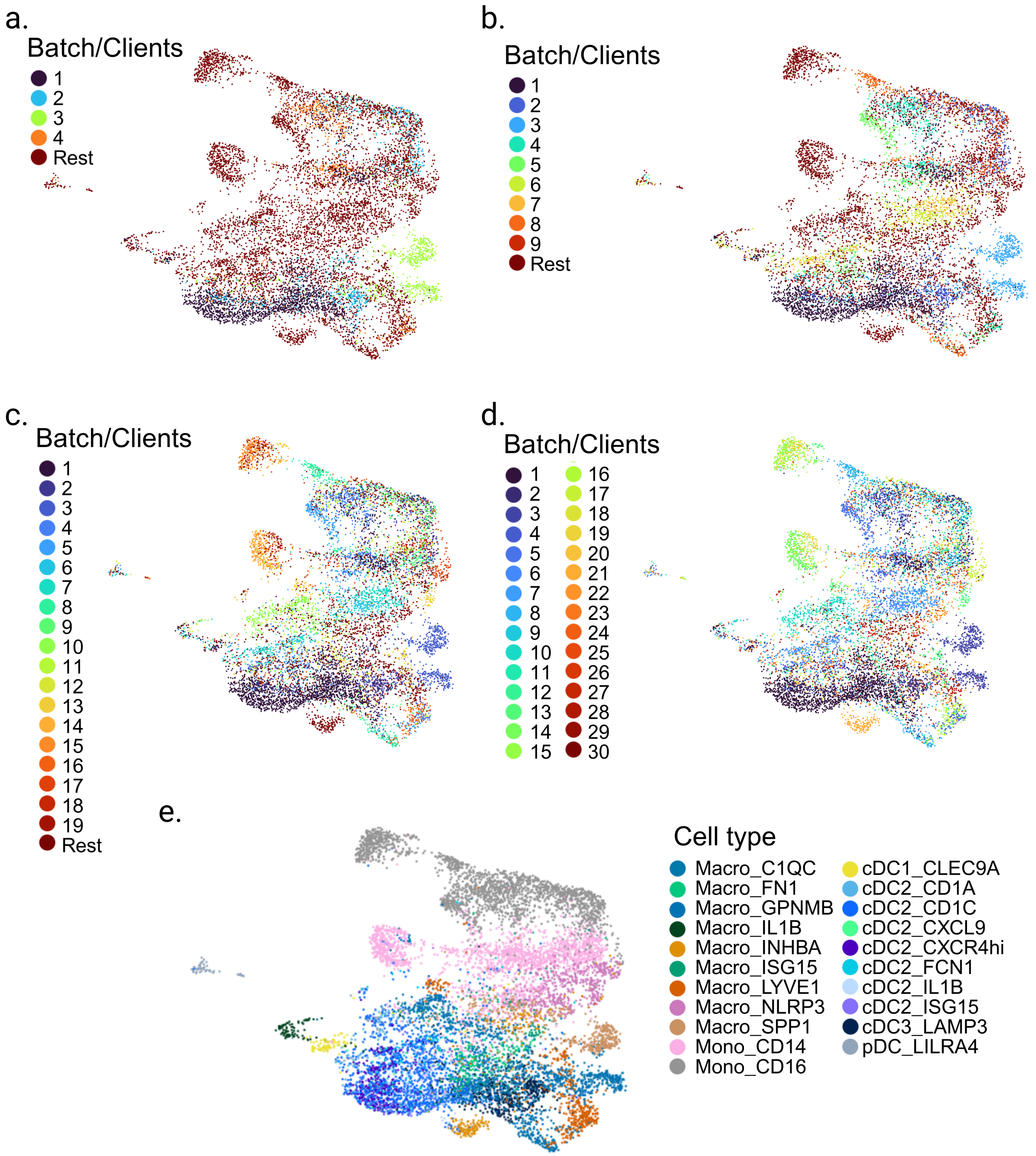

Fig S2: UMAP visualization of the Myeloid reference dataset across 30 batches and multiple cell types. In four federated scenarios, batches are sorted by size and assigned to clients: **a.** **Top5** – The four largest batches are assigned to clients 1–4, with the remaining batches grouped as client "rest." **b.** **Top10** – The nine largest batches are assigned to clients 1–9, with the remaining batches grouped as client "rest." **c.** **Top20** – The nineteen largest batches are assigned to clients 1–19, with the remaining batches grouped as client "rest." **d.** **Top30** – All thirty batches are treated as individual clients. **e.** UMAP of the Myeloid reference dataset annotated by known cell types.

2 Annotation

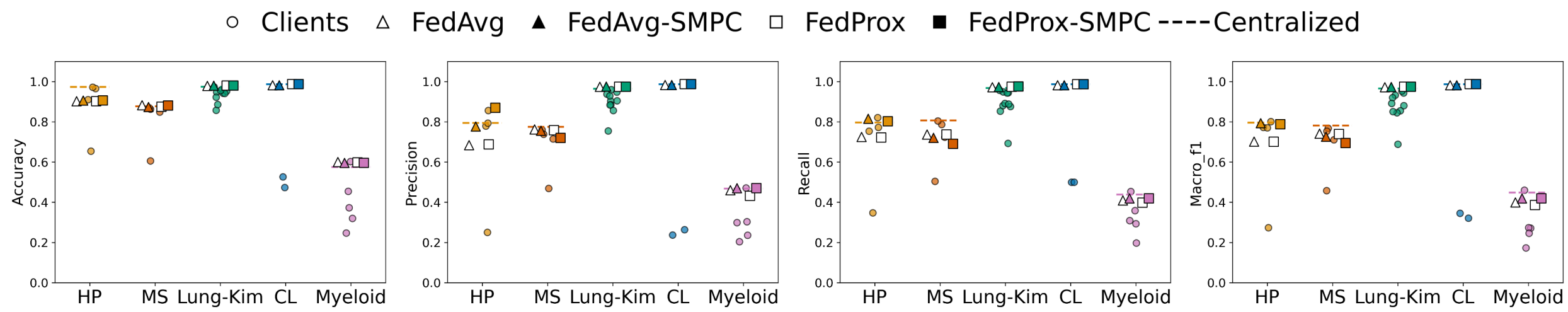

Fig S3: Performance comparison of the centralized model (dashed horizontal line), local client-level model (○), and federated models (FedAvg (△), FedAvg-SMPC (▲), FedProx (□), and FedProx-SMPC (■)) across Accuracy, Precision, Recall, and Macro-F1 (left to right) for cell type annotation on HP, MS, Lung-Kim, CL, and Myeloid (Top5) datasets. The centralized and client-level models were trained for 20 epochs, while the federated models were trained for 20 communication rounds with one local epoch per round.

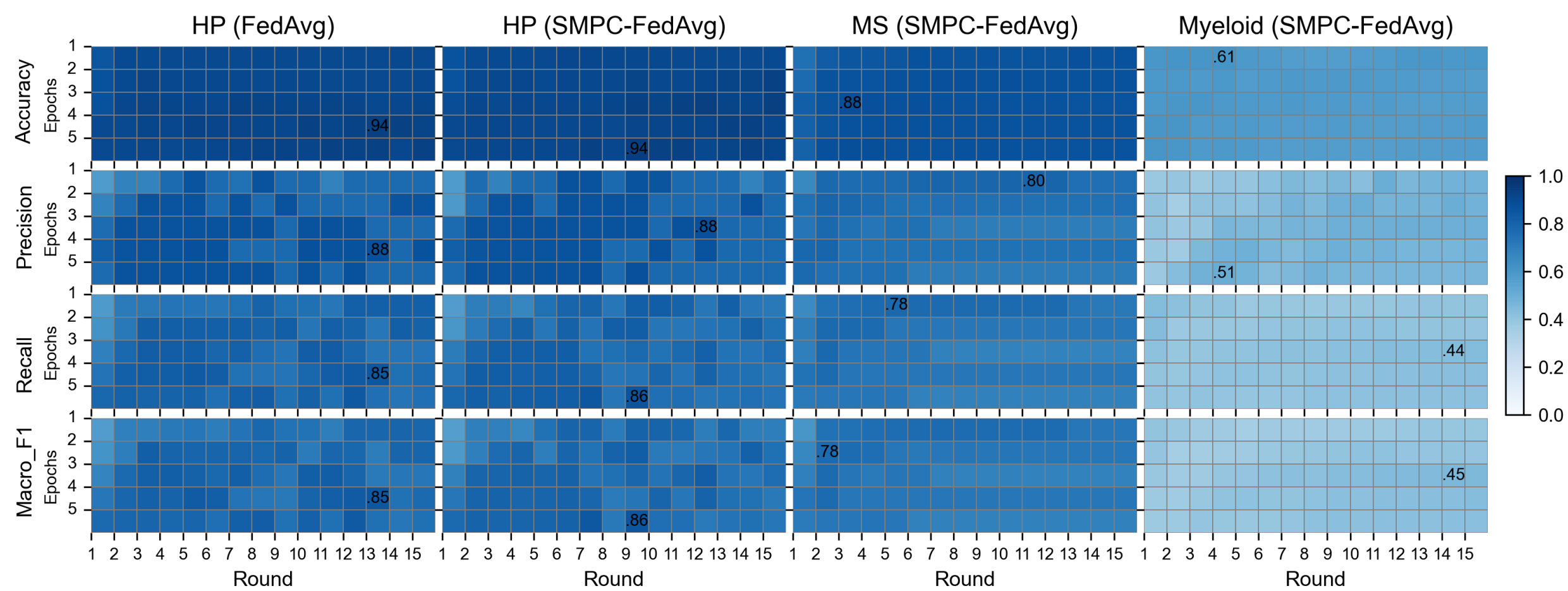

Fig S4: Performance heatmaps over 15 communication rounds and 1-5 local epochs for different federated aggregation methods and datasets. From left to right: HP (FedAvg), HP (SMPC-FedAvg), MS (SMPC-FedAvg), and Myeloid (SMPC-FedAvg). The effect of hyperparameters in each setting is evaluated in terms of Accuracy, Precision, Recall, and Macro-F1 (top to bottom).

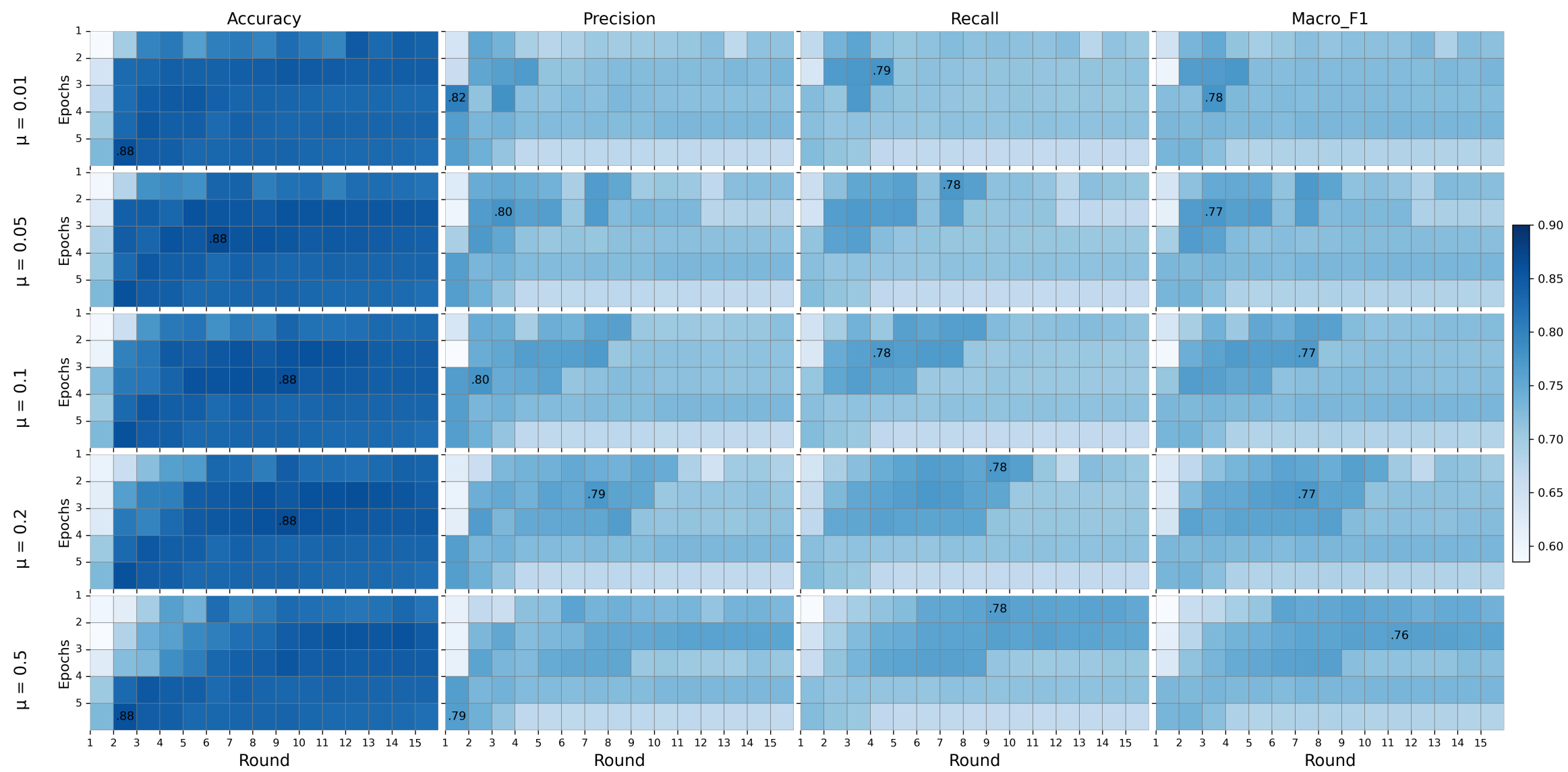

Fig S5: Performance of FedProx-SMPC across 15 communication rounds and 1-5 local epochs for different μ values ($\mu \in \{0.01, 0.05, 0.1, 0.2, 0.5\}$; rows top to bottom) on the MS dataset, evaluated in terms of Accuracy, Precision, Recall, and Macro-F1 (columns left to right).

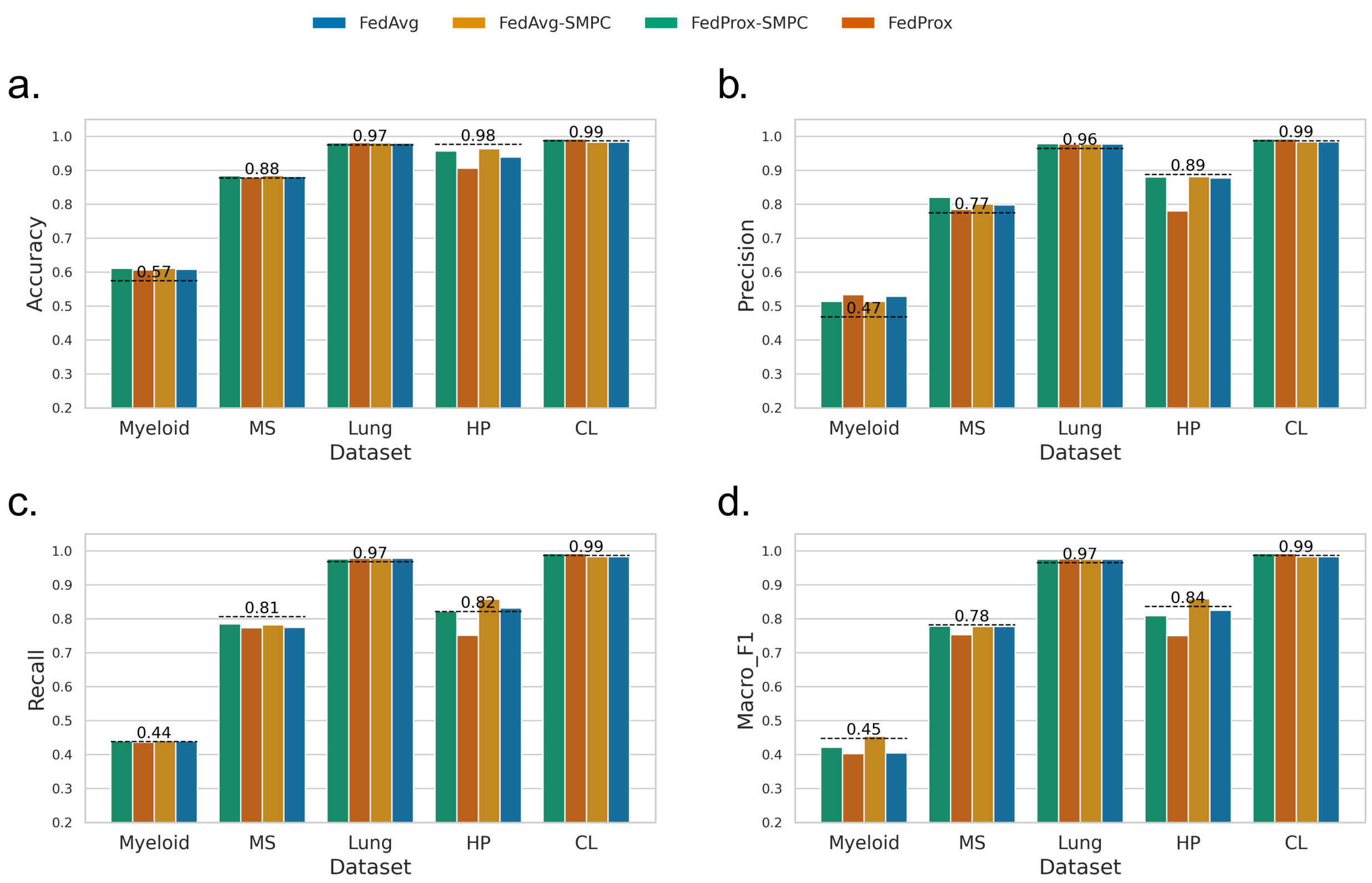

Fig S6: Best performance achieved by FedAvg and FedProx aggregation strategies, with and without SMPC (i.e., FedAvg, FedAvg-SMPC, FedProx, and FedProx-SMPC), across the Myeloid (Top5), MS, Lung, HP, and CL datasets. Dashed lines indicate centralized (non-federated) performance for each dataset and metric. **a.** Accuracy, **b.** Precision, **c.** Recall, and **d.** Macro-F1.

a. **Accuracy**

Dataset (Aggregation)	Best Value	n_epochs	Round
MS (FedProx-SMPC, μ=0.05)	0.884	3	6
MS (FedAvg)	0.882	1	20
MS (FedAvg-SMPC)	0.885	3	3
MS (FedProx, μ=0.01)	0.88	1	9
CL (FedProx-SMPC, μ=0.01)	0.992	1	7
CL (FedAvg)	0.983	1	7
CL (FedAvg-SMPC)	0.983	1	7
CL (FedProx, μ=0.01)	0.992	1	7
Lung (FedProx-SMPC, μ=0.01)	0.981	1	6
Lung (FedAvg)	0.98	1	11
Lung (FedAvg-SMPC)	0.981	1	13
Lung (FedProx, μ=0.01)	0.982	1	6
Myeloid (FedProx-SMPC, μ=0.01)	0.611	1	4
Myeloid (FedAvg)	0.608	1	3
Myeloid (FedAvg-SMPC)	0.611	1	4
Myeloid (FedProx, μ=0.01)	0.607	1	4
HP (FedProx-SMPC, μ=0.01)	0.956	1	52
HP (FedAvg)	0.938	3	17
HP (FedAvg-SMPC)	0.963	1	156
HP (FedProx, μ=0.01)	0.906	1	17

b. **Precision**

Dataset (Aggregation)	Best Value	n_epochs	Round
MS (FedProx-SMPC, μ=0.01)	0.82	3	1
MS (FedAvg)	0.798	1	8
MS (FedAvg-SMPC)	0.801	1	11
MS (FedProx, μ=0.01)	0.785	1	2
CL (FedProx-SMPC, μ=0.01)	0.992	1	7
CL (FedAvg)	0.983	1	7
CL (FedAvg-SMPC)	0.984	1	7
CL (FedProx, μ=0.01)	0.992	1	7
Lung (FedProx-SMPC, μ=0.01)	0.978	1	2
Lung (FedAvg)	0.978	1	2
Lung (FedAvg-SMPC)	0.978	1	11
Lung (FedProx, μ=0.01)	0.977	1	2
Myeloid (FedProx-SMPC, μ=0.01)	0.514	1	16
Myeloid (FedAvg)	0.528	1	19
Myeloid (FedAvg-SMPC)	0.514	1	16
Myeloid (FedProx, μ=0.01)	0.533	1	19
HP (FedProx-SMPC, μ=0.01)	0.88	1	53
HP (FedAvg)	0.877	3	17
HP (FedAvg-SMPC)	0.881	1	45
HP (FedProx, μ=0.01)	0.78	1	5

c. **Recall**

Dataset (Aggregation)	Best Value	n_epochs	Round
MS (FedProx-SMPC, μ=0.01)	0.785	2	4
MS (FedAvg)	0.775	1	8
MS (FedAvg-SMPC)	0.782	1	5
MS (FedProx, μ=0.01)	0.773	1	6
CL (FedProx-SMPC, μ=0.01)	0.992	1	7
CL (FedAvg)	0.982	1	1
CL (FedAvg-SMPC)	0.982	1	1
CL (FedProx, μ=0.01)	0.992	1	7
Lung (FedProx-SMPC, μ=0.01)	0.976	1	6
Lung (FedAvg)	0.978	1	7
Lung (FedAvg-SMPC)	0.978	1	7
Lung (FedProx, μ=0.01)	0.978	1	12
Myeloid (FedProx-SMPC, μ=0.01)	0.44	1	1
Myeloid (FedAvg)	0.44	1	1
Myeloid (FedAvg-SMPC)	0.441	3	17
Myeloid (FedProx, μ=0.01)	0.436	1	1
HP (FedProx-SMPC, μ=0.01)	0.822	3	6
HP (FedAvg)	0.831	3	11
HP (FedAvg-SMPC)	0.857	3	21
HP (FedProx, μ=0.01)	0.751	1	11

d. **Macro-F1**

Dataset (Aggregation)	Best Value	n_epochs	Round
MS (FedProx-SMPC, μ=0.01)	0.778	3	3
MS (FedAvg)	0.777	1	8
MS (FedAvg-SMPC)	0.777	2	2
MS (FedProx, μ=0.01)	0.753	1	6
CL (FedProx-SMPC, μ=0.01)	0.992	1	7
CL (FedAvg)	0.982	1	7
CL (FedAvg-SMPC)	0.983	1	7
CL (FedProx, μ=0.01)	0.992	1	7
Lung (FedProx-SMPC, μ=0.01)	0.975	1	6
Lung (FedAvg)	0.975	1	14
Lung (FedAvg-SMPC)	0.975	1	9
Lung (FedProx, μ=0.01)	0.976	1	14
Myeloid (FedProx-SMPC, μ=0.01)	0.422	1	19
Myeloid (FedAvg)	0.404	1	1
Myeloid (FedAvg-SMPC)	0.454	3	17
Myeloid (FedProx, μ=0.01)	0.403	1	1
HP (FedProx-SMPC, μ=0.01)	0.809	3	6
HP (FedAvg)	0.824	3	17
HP (FedAvg-SMPC)	0.858	3	21
HP (FedProx, μ=0.01)	0.75	1	11

Fig S7: Configurations of FedAvg, FedAvg-SMPC, FedProx, and FedProx-SMPC that achieved the best performance across the Myeloid (Top5), MS, Lung, HP, and CL datasets. For each aggregation strategy, the number of local epochs and communication rounds is reported alongside the corresponding performance value. For FedProx, the μ value is also indicated. **a.** Accuracy, **b.** Precision, **c.** Recall, **d.** Macro-F1.

Achieved accuracy in R rounds | E epochs | μ

a. Accuracy

<table>
<tr><th>Dataset (Aggregation)</th><th>70%</th><th>80%</th><th>90%</th><th>95%</th><th>99%</th></tr>
<tr><td>HP(FedAvg-SMPC)</td><td>1|1</td><td>1|1</td><td>1|4</td><td>7|5</td><td>NR</td></tr>
<tr><td>HP(FedProx-SMPC)</td><td>1|1|0.01</td><td>1|1|0.01</td><td>1|2|0.01</td><td>39|1|0.01</td><td>NR</td></tr>
<tr><td>MS(FedAvg-SMPC)</td><td>1|1</td><td>1|1</td><td>1|4</td><td>2|2</td><td>2|5</td></tr>
<tr><td>MS(FedProx-SMPC)</td><td>1|1|0.01</td><td>1|1|0.01</td><td>1|5|0.5</td><td>2|5|0.5</td><td>2|5|0.2</td></tr>
<tr><td>Myeloid(FedAvg-SMPC)</td><td>1|1</td><td>1|1</td><td>1|1</td><td>1|1</td><td>1|1</td></tr>
<tr><td>Myeloid(FedProx-SMPC)</td><td>1|1|0.01</td><td>1|1|0.01</td><td>1|1|0.01</td><td>1|1|0.01</td><td>1|1|0.01</td></tr>
</table>

b. Precision

<table>
<tr><th>Dataset (Aggregation)</th><th>70%</th><th>80%</th><th>90%</th><th>95%</th><th>99%</th></tr>
<tr><td>HP(FedAvg-SMPC)</td><td>1|1</td><td>1|3</td><td>1|3</td><td>1|3</td><td>1|4</td></tr>
<tr><td>HP(FedProx-SMPC)</td><td>1|1|0.01</td><td>1|2|0.01</td><td>2|3|0.01</td><td>2|3|0.01</td><td>3|3|0.01</td></tr>
<tr><td>MS(FedAvg-SMPC)</td><td>1|1</td><td>1|1</td><td>1|2</td><td>1|2</td><td>1|4</td></tr>
<tr><td>MS(FedProx-SMPC)</td><td>1|1|0.01</td><td>1|1|0.01</td><td>1|5|0.5</td><td>1|3|0.01</td><td>1|3|0.01</td></tr>
<tr><td>Myeloid(FedAvg-SMPC)</td><td>1|1</td><td>1|1</td><td>2|5</td><td>3|4</td><td>3|5</td></tr>
<tr><td>Myeloid(FedProx-SMPC)</td><td>1|1|0.01</td><td>1|1|0.01</td><td>6|1|0.01</td><td>7|1|0.01</td><td>11|1|0.01</td></tr>
</table>

c. Recall

<table>
<tr><th>Dataset (Aggregation)</th><th>70%</th><th>80%</th><th>90%</th><th>95%</th><th>99%</th></tr>
<tr><td>HP(FedAvg-SMPC)</td><td>1|1</td><td>1|3</td><td>1|5</td><td>1|5</td><td>2|4</td></tr>
<tr><td>HP(FedProx-SMPC)</td><td>1|3|0.01</td><td>1|2|0.01</td><td>2|3|0.01</td><td>2|3|0.01</td><td>3|3|0.01</td></tr>
<tr><td>MS(FedAvg-SMPC)</td><td>1|1</td><td>1|1</td><td>1|4</td><td>2|3</td><td>NR</td></tr>
<tr><td>MS(FedProx-SMPC)</td><td>1|1|0.01</td><td>1|1|0.01</td><td>1|3|0.01</td><td>2|2|0.05</td><td>NR</td></tr>
<tr><td>Myeloid(FedAvg-SMPC)</td><td>1|1</td><td>1|1</td><td>1|1</td><td>1|1</td><td>1|1</td></tr>
<tr><td>Myeloid(FedProx-SMPC)</td><td>1|1|0.01</td><td>1|1|0.01</td><td>1|1|0.01</td><td>1|1|0.01</td><td>1|1|0.01</td></tr>
</table>

d. Macro F1

<table>
<tr><th>Dataset (Aggregation)</th><th>70%</th><th>80%</th><th>90%</th><th>95%</th><th>99%</th></tr>
<tr><td>HP(FedAvg-SMPC)</td><td>1|1</td><td>1|3</td><td>1|4</td><td>1|5</td><td>2|3</td></tr>
<tr><td>HP(FedProx-SMPC)</td><td>1|3|0.01</td><td>1|2|0.01</td><td>2|3|0.01</td><td>2|3|0.01</td><td>4|3|0.01</td></tr>
<tr><td>MS(FedAvg-SMPC)</td><td>1|1</td><td>1|2</td><td>1|5</td><td>2|1</td><td>2|2</td></tr>
<tr><td>MS(FedProx-SMPC)</td><td>1|1|0.01</td><td>1|1|0.01</td><td>1|4|0.5</td><td>2|2|0.01</td><td>2|3|0.01</td></tr>
<tr><td>Myeloid(FedAvg-SMPC)</td><td>1|1</td><td>1|1</td><td>1|1</td><td>7|3</td><td>12|3</td></tr>
<tr><td>Myeloid(FedProx-SMPC)</td><td>1|1|0.01</td><td>1|1|0.01</td><td>1|1|0.01</td><td>NR</td><td>NR</td></tr>
</table>

Fig S8: Communication efficiency analysis of privacy-preserving federated aggregation strategies across the HP, MS, and Myeloid (Top5) datasets. The analysis shows the required communication rounds and local training epochs needed to reach 70%, 80%, 90%, 95%, and 99% of the centralized baseline performance. Each cell displays the number of communication rounds, local epochs, and μ value (only applicable for FedProx), separated by the symbol |. Entries marked as "NR" indicate that the corresponding performance threshold was not reached within the evaluated training budget. **a.** Accuracy, **b.** Precision, **c.** Recall, **d.** Macro-F1.

a. Accuracy

Dataset	Aggregation	Federated	Centralized	Difference
CL	FedProx-SMPC (E=1, R=7, Mu=0.01)	0.99	0.99	0.01
Covid-Corrected	FedAvg-SMPC (E=1, R=61)	0.93	0.92	0.01
HP	FedAvg-SMPC (E=1, R=156)	0.96	0.97	-0.01
Lung-Kim	FedAvg-SMPC (E=1, R=13)	0.98	0.97	0.01
MS	FedAvg-SMPC (E=3, R=3)	0.88	0.88	0.01
Myeloid-Top5	FedAvg-SMPC (E=1, R=4)	0.61	0.57	0.04
Myeloid-Top10	FedProx-SMPC (E=1, R=5, Mu=0.01)	0.61	0.57	0.03
Myeloid-Top20	FedProx-SMPC (E=1, R=55, Mu=0.01)	0.59	0.57	0.02
Myeloid-Top30	FedProx-SMPC (E=1, R=196, Mu=0.01)	0.56	0.57	-0.02

b. Precision

Dataset	Aggregation	Federated	Centralized	Difference
CL	FedProx-SMPC (E=1, R=7, Mu=0.01)	0.99	0.99	0.01
Covid-Corrected	FedAvg-SMPC (E=1, R=49)	0.59	0.6	-0.01
HP	FedAvg-SMPC (E=1, R=45)	0.88	0.79	0.09
Lung-Kim	FedProx-SMPC (E=1, R=2, Mu=0.01)	0.98	0.96	0.01
MS	FedProx-SMPC (E=3, R=1, Mu=0.01)	0.82	0.77	0.05
Myeloid-Top5	FedAvg-SMPC (E=1, R=16)	0.51	0.47	0.05
Myeloid-Top10	FedProx-SMPC (E=1, R=20, Mu=0.01)	0.51	0.47	0.05
Myeloid-Top20	FedProx-SMPC (E=1, R=35, Mu=0.01)	0.5	0.47	0.03
Myeloid-Top30	FedProx-SMPC (E=1, R=31, Mu=0.01)	0.49	0.47	0.03

c. Recall

Dataset	Aggregation	Federated	Centralized	Difference
CL	FedProx-SMPC (E=1, R=7, Mu=0.01)	0.99	0.99	0.01
Covid-Corrected	FedAvg-SMPC (E=1, R=83)	0.57	0.61	-0.04
HP	FedAvg-SMPC (E=3, R=21)	0.86	0.8	0.06
Lung-Kim	FedAvg-SMPC (E=1, R=7)	0.98	0.97	0.01
MS	FedProx-SMPC (E=2, R=4, Mu=0.01)	0.79	0.81	-0.02
Myeloid-Top5	FedAvg-SMPC (E=3, R=17)	0.44	0.44	0
Myeloid-Top10	FedProx-SMPC (E=1, R=2, Mu=0.10)	0.42	0.44	-0.02
Myeloid-Top20	FedProx-SMPC (E=1, R=38, Mu=0.01)	0.45	0.44	0.01
Myeloid-Top30	FedProx-SMPC (E=1, R=180, Mu=0.01)	0.46	0.44	0.02

d. Macro F1

Dataset	Aggregation	Federated	Centralized	Difference
CL	FedProx-SMPC (E=1, R=7, Mu=0.01)	0.99	0.99	0.01
Covid-Corrected	FedAvg-SMPC (E=1, R=83)	0.56	0.6	-0.03
HP	FedAvg-SMPC (E=3, R=21)	0.86	0.79	0.06
Lung-Kim	FedAvg-SMPC (E=1, R=9)	0.98	0.97	0.01
MS	FedProx-SMPC (E=3, R=3, Mu=0.01)	0.78	0.78	0
Myeloid-Top5	FedAvg-SMPC (E=3, R=17)	0.45	0.45	0.01
Myeloid-Top10	FedProx-SMPC (E=1, R=40, Mu=0.10)	0.42	0.45	-0.03
Myeloid-Top20	FedProx-SMPC (E=1, R=38, Mu=0.01)	0.46	0.45	0.01
Myeloid-Top30	FedProx-SMPC (E=1, R=186, Mu=0.01)	0.44	0.45	0

Fig S9: Performance comparison between the best privacy-preserving federated model and the centralized model for cell type annotation on the CL, Covid-corrected (Covid-19), HP, Lung-Kim, MS, and Myeloid datasets (all scenarios: Top5–Top30), evaluated across various metrics. All values are rounded to two decimal places for simplicity. **a.** Accuracy, **b.** Precision, **c.** Recall, **d.** Macro-F1.

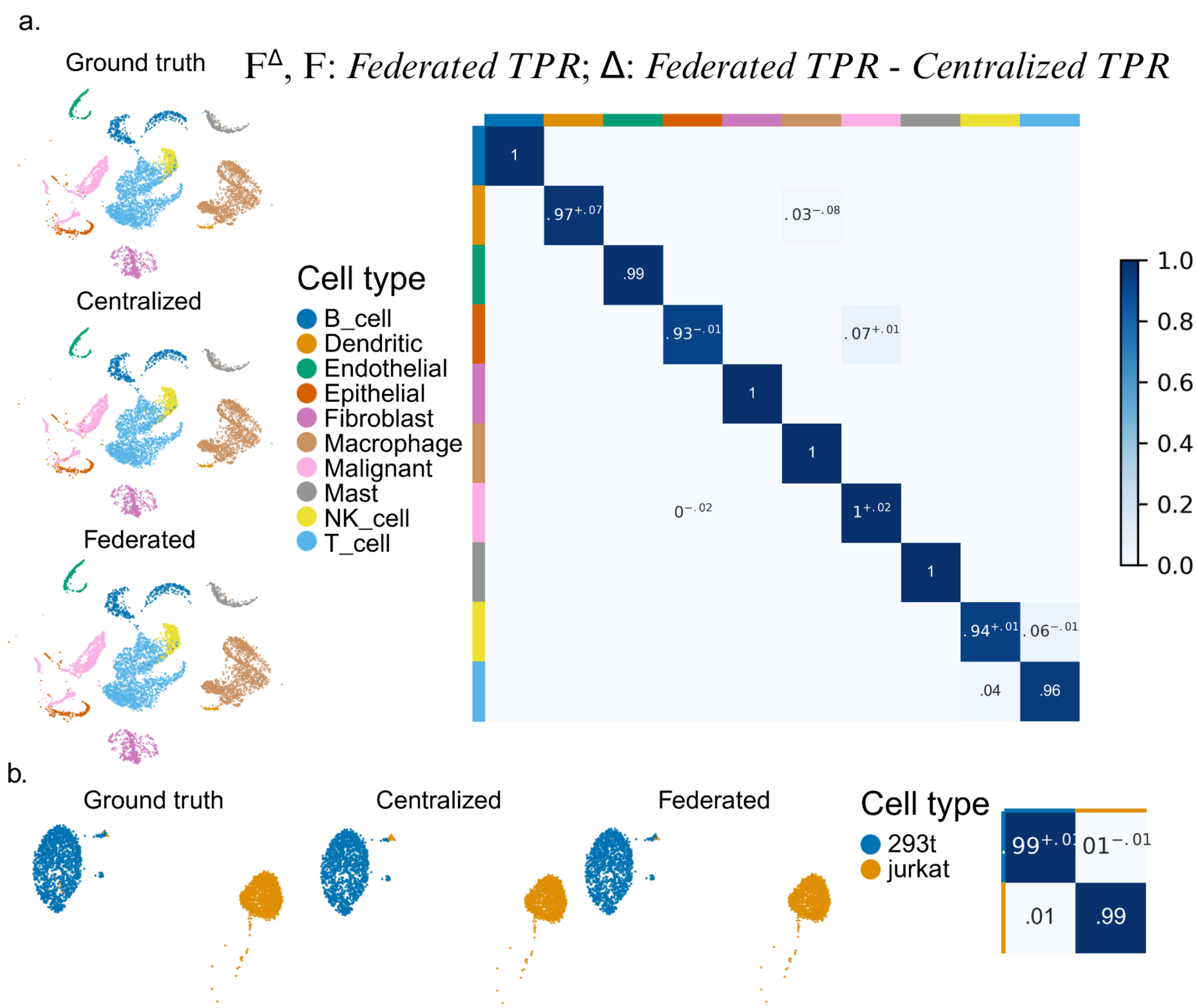

Fig S10: UMAP visualizations of ground truth, centralized predictions, and federated predictions of cell types, alongside confusion matrices showing federated performance in terms of per-class recall for each cell type. **a. Lung-Kim:** Federated results obtained using FedAvg-SMPC, trained for 1 local epoch over 7 communication rounds. **b. Cell Line (CL):** Results obtained using FedProx ($\mu = 0.01$), trained for 1 local epoch over 7 communication rounds.

3 Reference mapping

a. Accuracy

Dataset	Federated (SMPC)	Centralized	Difference
CL	0.97	0.97	0
Covid-Corrected	0.87	0.88	0
HP	0.85	0.85	0
Lung-Kim	0.95	0.95	0
MS	0.68	0.68	0.01
Myeloid-Top5	0.51	0.51	0
Myeloid-Top10	0.52	0.51	0.01
Myeloid-Top20	0.52	0.51	0.01
Myeloid-Top30	0.5	0.51	-0.01

b. Precision

Dataset	Federated (SMPC)	Centralized	Difference
CL	0.97	0.97	0
Covid-Corrected	0.57	0.59	-0.03
HP	0.7	0.69	0.01
Lung-Kim	0.95	0.95	0
MS	0.6	0.59	0.01
Myeloid-Top5	0.45	0.44	0.01
Myeloid-Top10	0.48	0.44	0.05
Myeloid-Top20	0.44	0.44	0.01
Myeloid-Top30	0.44	0.44	0.01

c. Recall

Dataset	Federated (SMPC)	Centralized	Difference
CL	0.97	0.97	0
Covid-Corrected	0.51	0.56	-0.05
HP	0.6	0.6	0
Lung-Kim	0.91	0.9	0.01
MS	0.56	0.55	0.01
Myeloid-Top5	0.37	0.37	0.01
Myeloid-Top10	0.37	0.37	0.01
Myeloid-Top20	0.38	0.37	0.01
Myeloid-Top30	0.36	0.37	-0.01

d. Macro F1

Dataset	Federated (SMPC)	Centralized	Difference
CL	0.97	0.97	0
Covid-Corrected	0.52	0.57	-0.04
HP	0.61	0.61	0
Lung-Kim	0.92	0.92	0
MS	0.56	0.55	0.01
Myeloid-Top5	0.39	0.38	0.01
Myeloid-Top10	0.39	0.38	0.01
Myeloid-Top20	0.39	0.38	0.01
Myeloid-Top30	0.37	0.38	-0.01

Fig S11: Performance comparison between the privacy-preserving federated reference mapping and the centralized reference mapping on CL, Covid-corrected (Covid-19), HP, Lung-Kim, MS, and Myeloid datasets (all scenarios: Top5–Top30), evaluated across various metrics. All values are rounded to two decimal places for simplicity. **a.** Accuracy, **b.** Precision, **c.** Recall, **d.** Macro-F1.

4 Scalability

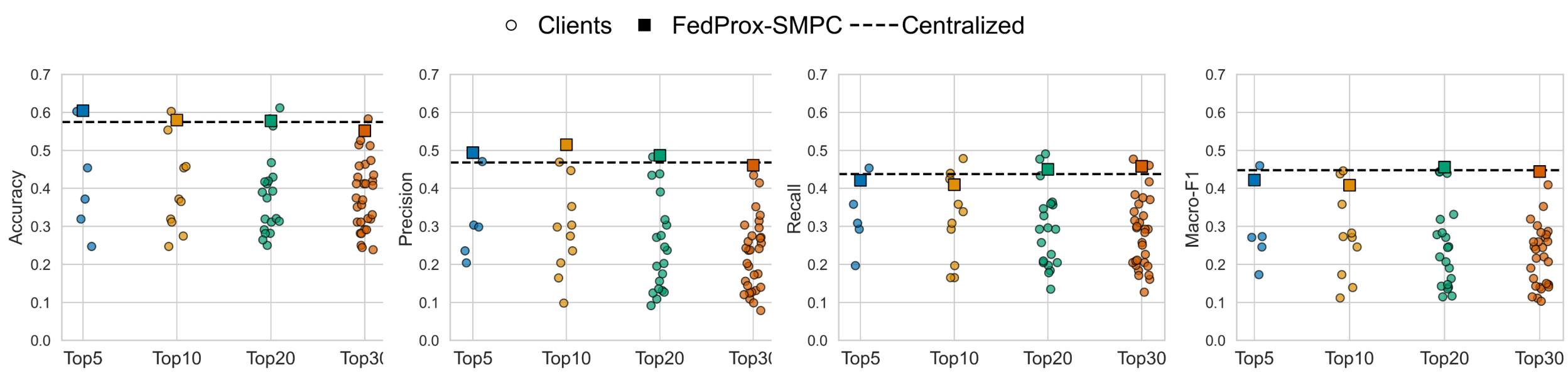

Fig S12: Scalability analysis of federated cell type annotation on the Myeloid dataset using FedProx-SMPC ($\mu = 0.01$) compared to the centralized model (dashed horizontal line) and the local client-level model (○), evaluated in terms of Accuracy, Precision, Recall, and Macro-F1 (left to right). Four scalability scenarios were designed with increasing numbers of clients (Top5, Top10, Top20, Top30). FedProx-SMPC was trained for 1 local epoch with varying numbers of communication rounds: 19 rounds for Top5, 20 for Top10, 38 for Top20, and 186 for Top30.

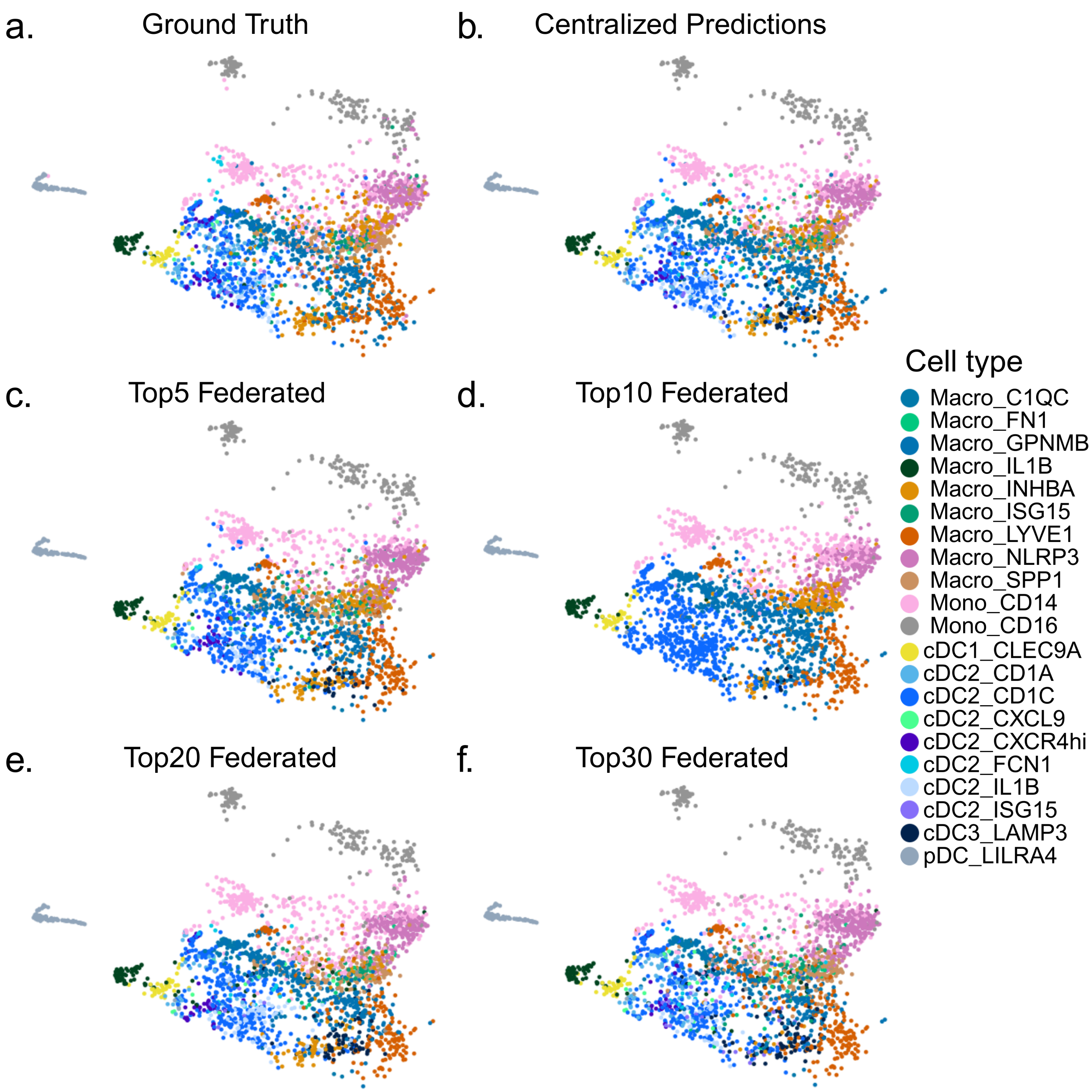

Fig S13: UMAP visualizations of ground truth cell types compared to predictions by the centralized model and FedProx-SMPC ($\mu = 0.01$) across various scalability scenarios of the Myeloid dataset. FedProx-SMPC was trained for one local epoch. **a.** Ground truth. **b.** Centralized: trained for 20 epochs. **c.** Top5: FedProx-SMPC trained for 19 communication rounds. **d.** Top10: FedProx-SMPC trained for 20 communication rounds. **e.** Top20: FedProx-SMPC trained for 38 communication rounds. **f.** Top30: FedProx-SMPC trained for 186 communication rounds.

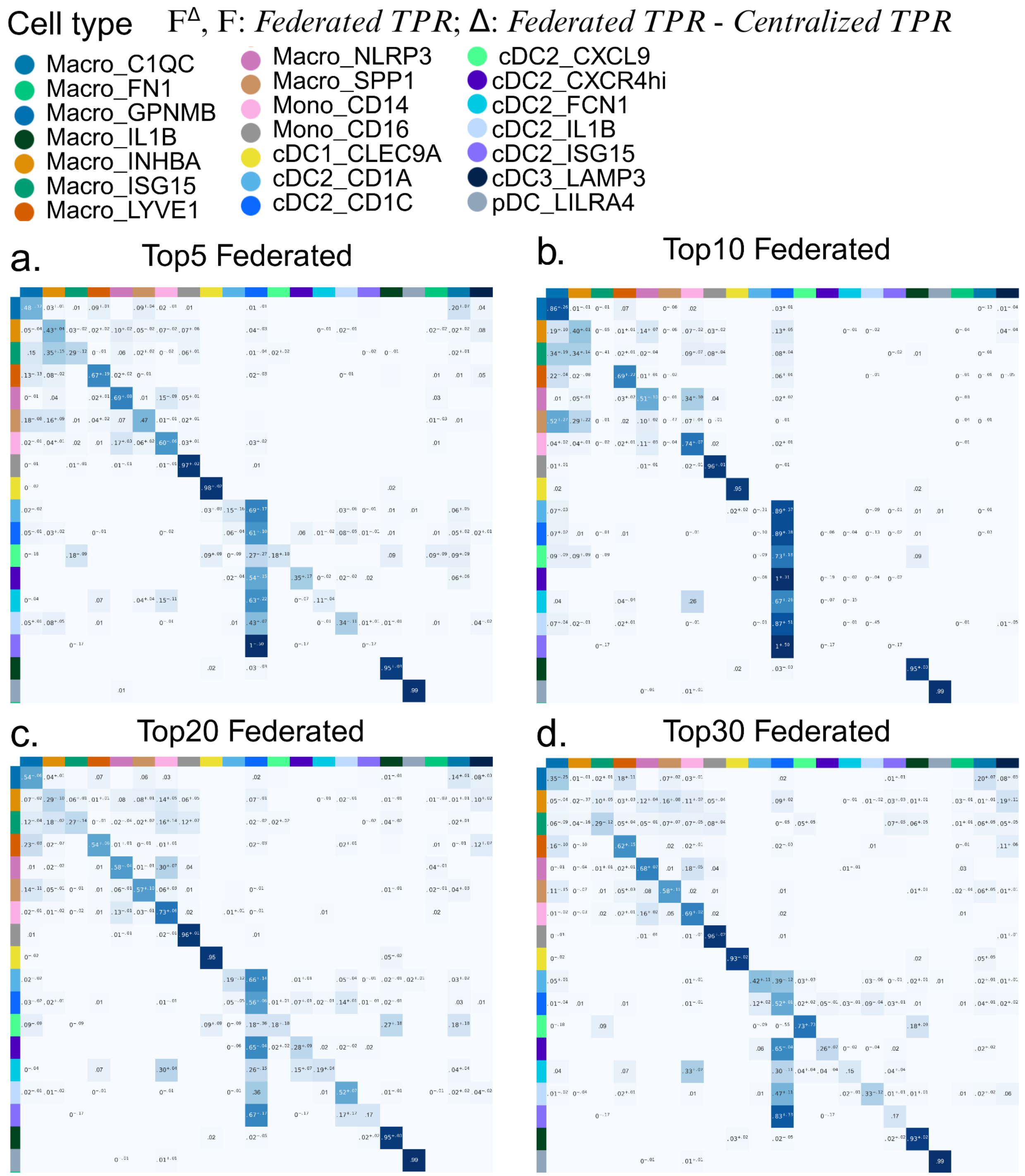

Fig S14: Confusion matrices of FedProx-SMPC ($\mu = 0.01$, trained for one local epoch) across various scalability scenarios of the Myeloid dataset. Each row represents the true cell type, and each column represents the predicted label. Per-class recall values are annotated, with superscripted differences relative to the centralized model. A plus sign indicates recall improvement, and a minus sign indicates a decrease. **a. Top5** (19 communication rounds). **b. Top10** (20 rounds). **c. Top20** (38 rounds). **d. Top30** (186 rounds).

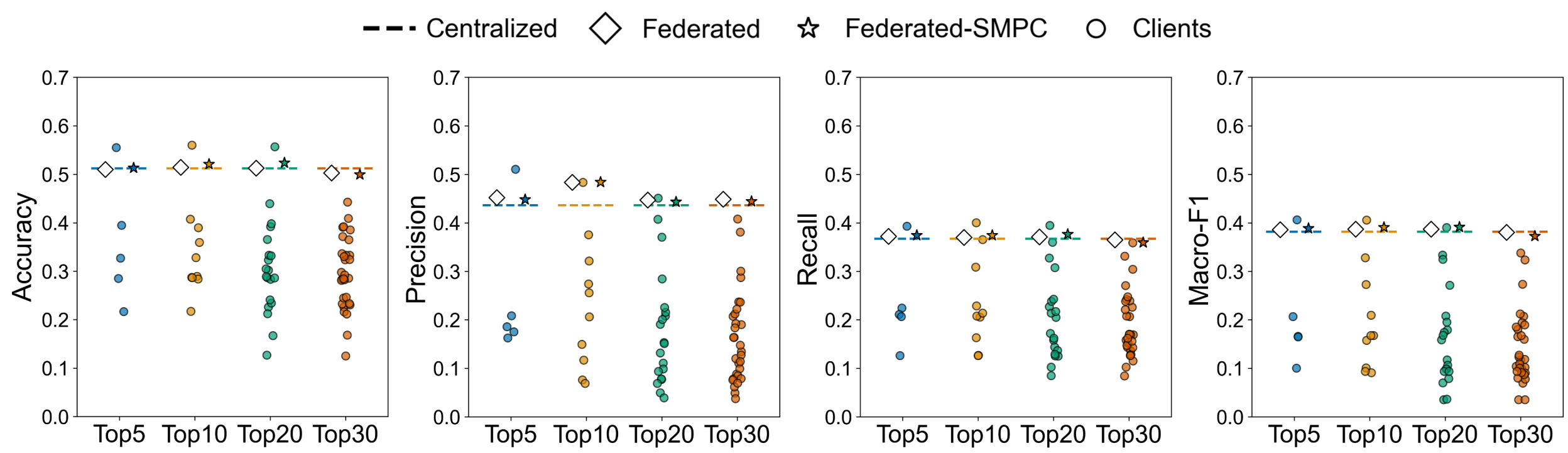

Fig S15: Performance comparison between the privacy-preserving federated models (Federated (⋄) and Federated-SMPC (⋆)), the centralized model (dashed horizontal line), and the local client-level model (∘) for reference mapping across various scalability scenarios of the Myeloid dataset, evaluated in terms of Accuracy, Precision, Recall, and Macro-F1 (left to right).

5 Batch effect

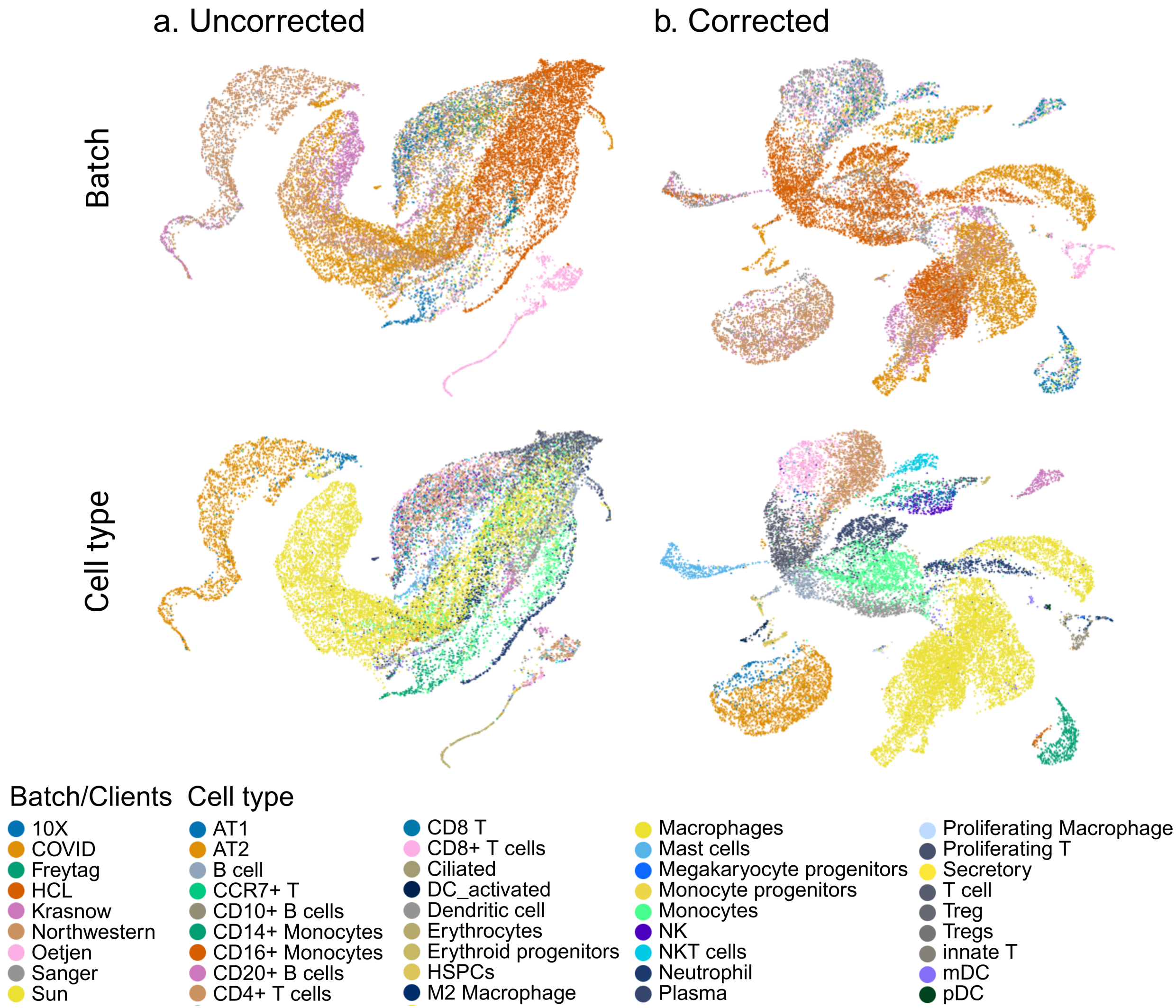

Fig S16: UMAP visualizations of cell types and batches in the COVID-19 dataset. **a.** Uncorrected COVID-19 dataset. **b.** Batch-effect corrected COVID-19 dataset using the scGen model.

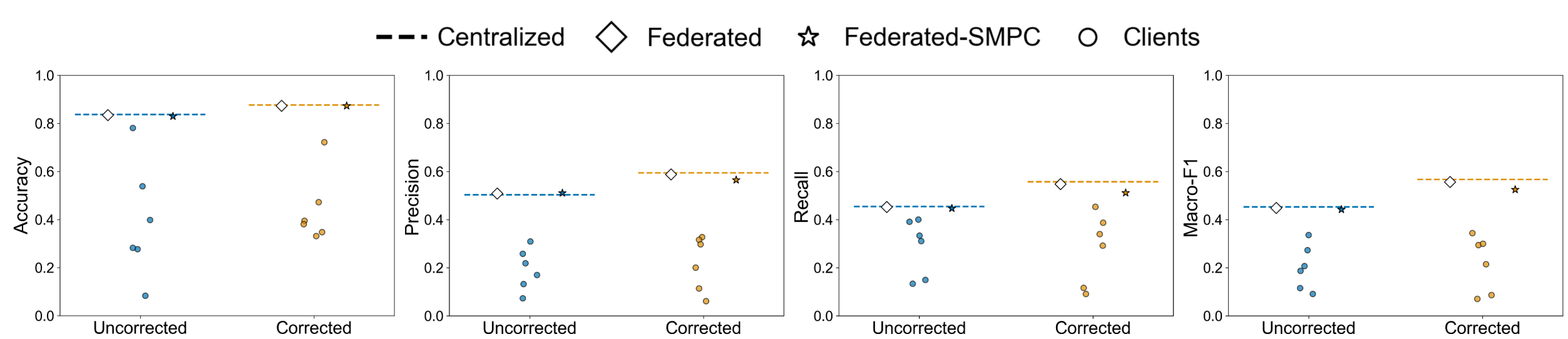

Fig S17: Performance comparison between the privacy-preserving federated models (Federated (◇) and Federated-SMPC (⋆)), the centralized model (dashed horizontal line), and the local client-level model (○) for reference mapping of the COVID-19 dataset, evaluated in terms of Accuracy, Precision, Recall, and Macro-F1 (left to right).

6 Federated binning benchmark

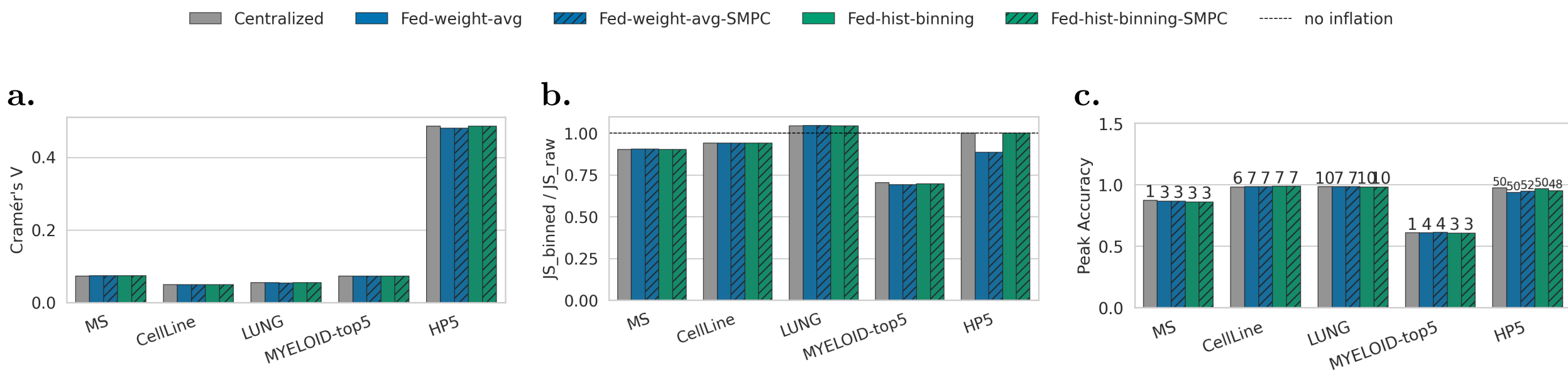

Fig S18: Comparison of local non-federated scGPT binning and federated Clifti-GPT binning strategies across the five scGPT annotation datasets (MS, CellLine, LUNG, MYELOID-top5, and HP5). For each dataset, cells were partitioned into federated clients using the dataset-specific batch column. Expression bin edges were computed using five binning strategies: local quantile binning independently on each client's data, federated weighted-average binning, SMPC-protected federated weighted-average binning, federated histogram binning, and SMPC-protected federated histogram binning. Grey bars denote local non-federated scGPT binning, and hatching indicates the SMPC privacy-preserving variants of the federated binning strategies. **a.** Cramér's V between client identity and bin assignment after binning. Lower values indicate that bin assignments are weakly associated with client identity, whereas higher values indicate stronger client-specific binning artifacts. **b.** Heterogeneity amplification, defined as $\mathrm{JS}_{\mathrm{binned}}/\mathrm{JS}_{\mathrm{raw}}$, where $\mathrm{JS}_{\mathrm{raw}}$ is the mean pairwise Jensen–Shannon divergence among client expression distributions on raw non-zero values and $\mathrm{JS}_{\mathrm{binned}}$ is the corresponding divergence after discretization. The dashed line marks a ratio of 1; values above this line indicate that binning increases apparent cross-client heterogeneity, whereas values at or below 1 indicate no inflation. **c.** Clifti-GPT cell-type annotation accuracy obtained with each binning strategy. The best communication round for each dataset–strategy pair is annotated above the corresponding bar.

7 Communication cost

Table 1: Fine-tuning weight sharing: measured wall-clock. Fixed $P = 3$ SMPC parties.

Mode	C	Configuration	t (median)	Overhead
Plaintext	2		289 μs	1.0×
SMPC	2		32.45 ms	112.2×
Plaintext	3		721 μs	1.0×
SMPC	3	$\lvert\theta\rvert = 10^6$	31.41 ms	43.5×
Plaintext	5		657 μs	1.0×
SMPC	5		35.30 ms	53.7×
Plaintext	2		2.04 ms	1.0×
SMPC	2		112.91 ms	55.5×
Plaintext	3		3.03 ms	1.0×
SMPC	3	$\lvert\theta\rvert = 10^7$	119.96 ms	39.5×
Plaintext	5		4.97 ms	1.0×
SMPC	5		119.21 ms	24.0×
Plaintext	2		10.19 ms	1.0×
SMPC	2		502.58 ms	49.3×
		$\lvert\theta\rvert = 51337228$		

Mode	C	Configuration	t (median)	Overhead
Plaintext	3		14.89 ms	1.0×
SMPC	3		504.24 ms	33.9×
Plaintext	5		24.43 ms	1.0×
SMPC	5		532.57 ms	21.8×

Table 2: Federated reference mapping (KNN): measured wall-clock. Fixed $P = 3$ SMPC parties.

Mode	C	Configuration	t (median)	Overhead
Plaintext	2	$n_q = 2000$	2.98 ms	1.0×
SMPC	2	$n_r = 1000$	56.68 s	19047.2×
Plaintext	5	$d = 128$	3.52 ms	1.0×
SMPC	5	$k = 10$	82.50 s	23420.5×
Plaintext	2	$n_q = 2000$	2.87 ms	1.0×
SMPC	2	$n_r = 1000$	27.94 s	9749.3×
Plaintext	5	$d = 128$	3.39 ms	1.0×
SMPC	5	$k = 5$	37.67 s	11112.1×
Plaintext	2	$n_q = 2000$	5.99 ms	1.0×
SMPC	2	$n_r = 10^4$	419.65 s	70094.1×
Plaintext	5	$d = 128$	6.98 ms	1.0×
SMPC	5	$k = 10$	450.68 s	64611.3×
Plaintext	2	$n_q = 2000$	11.15 ms	1.0×
SMPC	2	$n_r = 10^4$	211.55 s	18968.7×
Plaintext	5	$d = 128$	11.73 ms	1.0×
SMPC	5	$k = 5$	230.18 s	19620.7×
Plaintext	2	$n_q = 2000$	6.44 ms	1.0×
SMPC	2	$n_r = 5000$	220.07 s	34160.1×
Plaintext	5	$d = 128$	7.19 ms	1.0×
SMPC	5	$k = 10$	250.64 s	34875.2×
Plaintext	2	$n_q = 2000$	6.07 ms	1.0×
SMPC	2	$n_r = 5000$	118.01 s	19453.0×
Plaintext	5	$d = 128$	6.63 ms	1.0×
SMPC	5	$k = 5$	124.65 s	18791.2×
Plaintext	2	$n_q = 500$	997 μs	1.0×
SMPC	2	$n_r = 1000$	35.43 s	35553.4×
Plaintext	5	$d = 128$	1.33 ms	1.0×
SMPC	5	$k = 10$	66.05 s	49554.7×
Plaintext	2	$n_q = 500$	955 μs	1.0×
SMPC	2	$n_r = 1000$	17.60 s	18427.5×
Plaintext	5	$d = 128$	1.30 ms	1.0×
SMPC	5	$k = 5$	31.52 s	24174.0×
Plaintext	2	$n_q = 500$	2.52 ms	1.0×
SMPC	2	$n_r = 10^4$	112.74 s	44688.3×
Plaintext	5	$d = 128$	5.58 ms	1.0×
SMPC	5	$k = 10$	152.12 s	27249.9×

Mode	C	Configuration	t (median)	Overhead
Plaintext	2	$n_q = 500$	2.99 ms	1.0×
SMPC	2	$n_r = 10^4$	58.52 s	19575.5×
Plaintext	5	$d = 128$	5.45 ms	1.0×
SMPC	5	$k = 5$	66.47 s	12194.7×
Plaintext	2	$n_q = 500$	2.77 ms	1.0×
SMPC	2	$n_r = 5000$	73.46 s	26476.0×
Plaintext	5	$d = 128$	4.06 ms	1.0×
SMPC	5	$k = 10$	102.24 s	25194.3×
Plaintext	2	$n_q = 500$	2.62 ms	1.0×
SMPC	2	$n_r = 5000$	37.02 s	14114.2×
Plaintext	5	$d = 128$	3.94 ms	1.0×
SMPC	5	$k = 5$	52.60 s	13338.7×

Table 3: Federated binning: measured wall-clock. Fixed $P = 3$ SMPC parties.

Mode	C	Configuration	t (median)	Overhead
W-avg	2		280 μs	1.0×
W-avg SMPC	2		3.92 ms	14.0×
Hist	2		989 μs	1.0×
Hist SMPC	2		9.06 ms	9.2×
W-avg	5		1.78 ms	1.0×
W-avg SMPC	5	$B = 51$	5.03 ms	2.8×
Hist	5	$M = 4096$	1.40 ms	1.0×
Hist SMPC	5		11.95 ms	8.6×
W-avg	10		1.88 ms	1.0×
W-avg SMPC	10		9.02 ms	4.8×
Hist	10		751 μs	1.0×
Hist SMPC	10		18.75 ms	25.0×

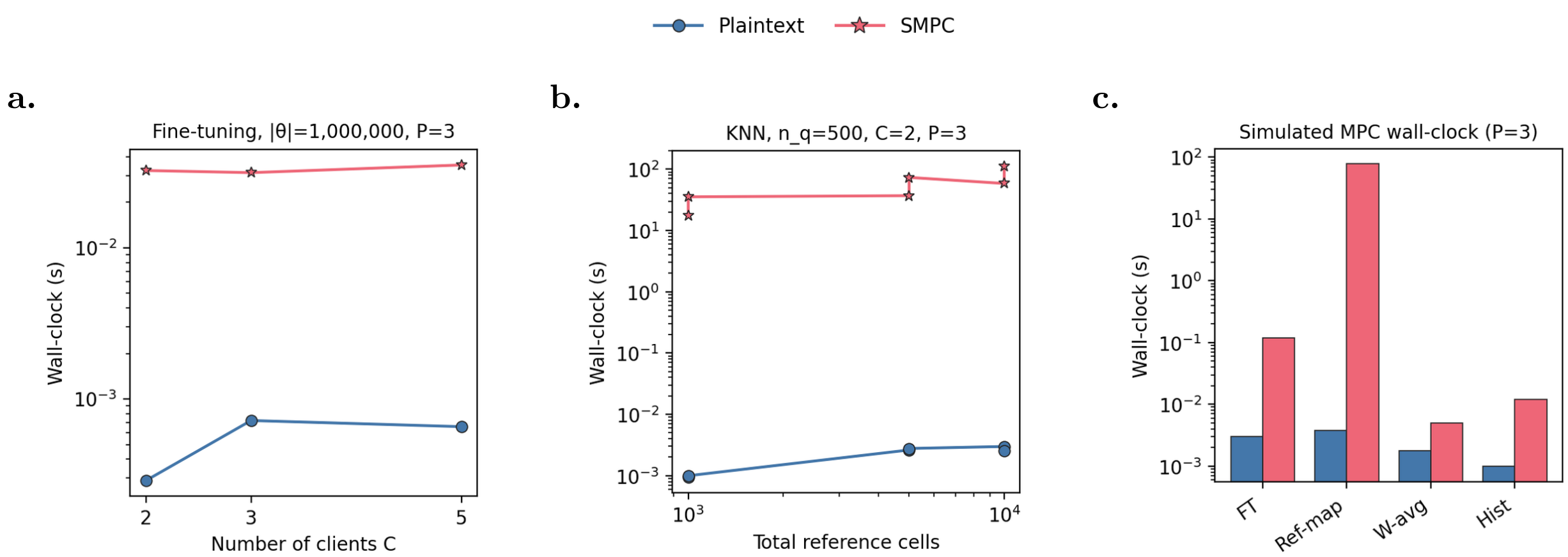

Fig S19: Communication-cost benchmark for the federated Clifti-GPT workflows, comparing plaintext (○) and CrypTen SMPC (⋆) with $P = 3$ GPU parties. **a.** Median measured wall-clock for one fine-tuning aggregation as a function of federated clients C, holding trainable parameters $|\theta|$ fixed (log–log scale). **b.** Median measured wall-clock for the federated reference-mapping (KNN) pass as a function of total reference size, holding n_q and C fixed (log–log scale). **c.** Median measured wall-clock aggregated over the evaluated configuration grid for fine-tuning aggregation, reference mapping, weighted-average binning, and histogram binning. Timings were profiled on GPU with simulated CrypTen MPC and exclude real inter-site network latency.
